# Effectiveness of structured lactation support and human donor milk banking in German NICUs: a stepped-wedge cluster-randomized trial

**DOI:** 10.1186/s12916-026-05211-1

**Published:** 2026-09-11

**Authors:** Juliane Köberlein-Neu, Nicola Gabriela Dymek, Viola Zimmer, Trutz Bommhardt, Anna Katharina Stirner, Isabella Güldenring, Tim Ohnhäuser, Daniel Wiesen, Till Dresbach, Nadine Scholten, Neo-MILK Consortium/collaborators

**Affiliations:** 1 Centre for Health Economics and Health Services Research, University of Wuppertal, Rainer-Gruenter-Str. 21, 42119 Wuppertal, Germany; 2 Department of Business Administration and Health Care Management, University of Cologne, Cologne, Germany; 3 Department for Psychosomatic Medicine and Psychotherapy, Centre for Health Services Research and Communication (CHSR), University Hospital Bonn, Bonn, Germany; 4 Faculty of Medicine and University Hospital Cologne, Institute of Medical Sociology, Health Services Research and Rehabilitation Science, Chair of Health Services Research, University of Cologne, Cologne, Germany; 5 Erasmus School of Health Policy and Management (ESHPM), Erasmus University Rotterdam, Rotterdam, Netherlands; 6 Department of Neonatology, University Hospital Bonn, Bonn, Germany

**Keywords:** Lactation support, Human donor milk, Very low birth weight infants, Stepped-wedge cluster-randomized trial, Hybrid effectiveness-implementation trial, Mother’s own milk feeding, Neonatal care

## Abstract

**Background:**

Although exclusive human milk feeding is beneficial for very low birth weight infants, rates remain suboptimal globally. This hybrid type 1 effectiveness-implementation trial evaluated structured lactation support combined with human donor milk banking in neonatal intensive care units (NICUs), primarily assessing effectiveness, with secondary assessment of implementation and economic outcomes.

**Methods:**

We conducted a stepped-wedge cluster-randomized trial in 15 German Level I NICUs (April 2022–March 2024). The Neo-MILK intervention comprised structured lactation counselling, multilingual materials, mobile app support for mothers/parents, and human donor milk banking. Implementation strategies included video-based staff training, guidelines, commitment nudges for medical staff, and support for human donor milk bank establishment. The primary outcome was exclusive mother’s own milk feeding at NICU discharge. Secondary outcomes included feeding status categories, clinical complications, length of stay, and maternal outcomes. Implementation outcomes were acceptability, feasibility, appropriateness, fidelity, and sustainability. Prespecified subgroup analyses examined birth weight (< 1,000 g vs. ≥1,000 g) and illness severity (CRIB ≥ 11 vs. <11). Economic evaluation employed time-driven activity-based costing and budget impact analysis.

**Results:**

Among 1,627 very low birth weight infants (831 intervention, 796 control), the intervention increased exclusive mother’s own milk feeding at discharge by 11%-points (95% CI: 3.0 to 19.0; *p* = 0.007) from a 43% baseline. Exclusive formula feeding decreased correspondingly. Subgroup analyses showed no significant effects in extremely low birth weight infants (< 1,000 g) or high-risk infants (CRIB ≥ 11). Clinical complications and length of stay showed no differences. Acceptability, appropriateness, and feasibility were high. Fidelity was sufficient, with prepartal counselling delivered as intended in 71% of cases. Human donor milk banks were established in 40% of sites. Sustainability indicators were promising, with 61% of staff reporting routine integration. Economic evaluation suggested potential net savings nationally in the base-case scenario.

**Conclusions:**

Structured lactation support with human donor milk banking increased exclusive mother’s own milk feeding across Level I NICUs, with no observed increase in complications or costs. Implementation appeared feasible, with indications of sustainability. Variable uptake and differential effects in highest-risk subgroups highlight the need for ongoing organizational support and tailored strategies to further optimize effectiveness.

**Clinical trial registration:**

German Clinical Trials Register DRKS00025058.

**Supplementary Information:**

The online version contains supplementary material available at https://doi.org/10.1186/s12916-026-05211-1.

## Background

Human milk (HM) provides important nutritional benefits for very low birth weight (VLBW) infants and is associated with reduced risks of necrotizing enterocolitis, late-onset sepsis, retinopathy of prematurity, bronchopulmonary dysplasia, and with more favourable neurodevelopmental outcomes [1–11]. The World Health Organization (WHO) recommends mother’s own milk (MOM) from the first day of life. In cases where MOM is not or only partially available, the WHO advises using human donor milk (HDM) as an alternative or a supplement to MOM [12].

Despite the compelling evidence of MOM’s essential role, the rate of VLBW infants exclusively fed with MOM at discharge from the neonatal intensive care unit (NICU) remains suboptimal. International studies have reported exclusive MOM feeding rates at discharge between 30% and 65% [13–15], far below rates in term infants [16]. This persistent evidence–practice gap points to fundamental challenges in translating clinical evidence into sustainable real-world practice and underscores the need for multi-site evaluations of scalable interventions.

Recent systematic reviews have examined lactation interventions and human donor milk banks (HDMBs) [16, 17]. Song et al.’s meta-analysis reported that comprehensive policy packages including breastfeeding support programs, kangaroo mother care, and supportive NICU policies, were associated with higher exclusive breastfeeding rates (including expressed milk feeding) upon hospital or NICU discharge. However, there was considerable heterogeneity in intervention types, study designs and outcomes [16]. Williams et al.’s systematic review of HDM programs found mixed evidence regarding effects on breastfeeding rates, attributed to heterogeneous populations, varied implementation approaches, and methodological limitations, including that only two of 10 studies enrolled participants from more than one hospital [17]. Both syntheses reveal critical gaps that constrain the ability to translate promising interventions into routine practice across diverse healthcare settings.

Firstly, the predominance of single-centre research raises questions about transferability across diverse organizational contexts. Most studies were conducted in single institutions [17], limiting understanding of whether interventions remain effective when transferred to settings with different contextual characteristics. This is a limitation that extends beyond HDM research to the broader field of NICU lactation interventions [18, 19]. Research on complex interventions emphasizes that effectiveness in routine care depends on organizational readiness, leadership support, staff capability, and workflow alignment [20, 21].

Secondly, while lactation-support interventions and HDM banking show promise individually, there is limited evidence on integrated models that combine both as complementary components. In particular, Song et al. note that studies including HDMBs rarely articulated a rationale for how HDM availability might influence maternal lactation outcomes [16]. Williams et al.’s review identified the same gap, finding that inconsistent effects may stem from how HDM programs are conceptualized and implemented [17]. Most studies treat HDMBs as standalone services rather than as integrated components [22]. Emerging evidence suggests that integrated HDM access can reinforce institutional commitment to HM feeding and provide a safety net that reduces provider and parental anxiety [17, 23–25]. Concerns have been raised, however, that HDM availability may inadvertently discourage maternal breastfeeding [17]. Systematic evaluation of integrated models positioning HDM banking as a fundamental component of comprehensive lactation support remains scarce, particularly in pragmatic, multi-site trials reflecting real-world complexity.

Thirdly, there is limited evidence on whether lactation support interventions can be delivered consistently and sustained in routine clinical practice. Most studies report clinical outcomes without systematically assessing whether interventions were delivered as intended, whether staff adopted new practices, or whether improvements persisted beyond the study period [16, 26]. Commonly encountered practical barriers including low staff training uptake, competing organizational priorities, and resource constraints [27, 28] are well recognized but rarely addressed through structured strategies alongside effectiveness evaluation. As a result, clinicians and policymakers lack evidence on feasibility and sustainability when deciding whether to adopt such interventions.

To address these evidence gaps pragmatically through a multi-site approach [16, 17, 26], we developed and evaluated Neo-MILK (Structured Lactation Support and Establishment of Human Donor Milk Banks in Neonatal Centres). We designed a multi-component intervention integrating structured lactation support with HDMB establishment as a core component, building on the assumption that such integration may enhance effectiveness by reducing formula exposure and by creating mutually reinforcing systems that strengthen commitment to HM feeding. Following extensive interest-holder engagement with German neonatologists, nurses, lactation consultants, and mothers of VLBW infants to ensure contextual appropriateness [29], we employed a stepped-wedge cluster-randomized design across 15 diverse German Level I NICUs to evaluate intervention effectiveness while gathering information on implementation processes [30]. In Germany, Level I denotes the highest level of perinatal care, i.e. centres caring for the most immature and critically ill infants; this is the inverse of US usage, where Level I designates basic newborn care [31]. Although the study was conducted in Germany, the challenges it addressed, such as fragmented lactation support, resource constraints and inadequate HDM access, are widely reported in international literature [16, 17, 27], suggesting relevance beyond the specific study context.

This paper reports the effectiveness findings of the Neo-MILK stepped-wedge cluster-randomized trial, together with descriptive implementation and economic results. Following the study protocol [32], the trial addressed three objectives: (i) to compare the effectiveness of the Neo-MILK intervention components and their implementation strategies with the current standard of care, with exclusive MOM feeding at NICU discharge as the primary endpoint and major neonatal complications among the secondary endpoints; (ii) to describe the extent to which the intervention was delivered, adopted and sustained across the participating NICUs; (iii) to examine the resource requirements of the intervention and its implementation and to project the budgetary consequences of nationwide adoption. We hypothesized that this integrated, multi-component intervention would significantly improve exclusive MOM feeding rates at NICU discharge among VLBW infants compared to standard care. A further protocol objective, in particular the understanding for whom and under what circumstances the intervention components work, is addressed in a mixed-methods process evaluation reported separately.

## Methods

This trial was designed to evaluate whether a structured lactation support programme combined with the establishment of human donor milk banks increases exclusive MOM feeding at NICU discharge among VLBW infants, and to describe the implementation and resource requirements of that programme in routine care. Outcomes corresponding to these objectives are summarised in Table 1. We conducted a multi-centre, prospective, hybrid type 1 effectiveness-implementation cluster-randomized controlled trial in Level I NICUs accompanied by a mixed-methods process evaluation and an economic evaluation. The study used a stepped-wedge design involving a sequential crossover of clusters from the control to the intervention condition, with all clusters starting in the control condition. The stepped-wedge design was chosen because all NICUs wished to receive the intervention, which enhanced acceptability and ethical feasibility while providing efficient evaluation across fewer clusters than a parallel design [30]. NICUs were randomized to one of three sequences, dictating the timing of crossover to the intervention condition. The study included four measurement periods over 24 months, with 6-month intervals between steps, and a subsequent two-month follow-up. Each cluster contributed data during both control and intervention periods, with measurement occurring continuously throughout each period using a cross-sectional sampling approach where different participants were assessed at each timepoint.

**Table 1:** Summary of effectiveness and implementation outcomes

Outcome	Indicator	Timepoint	Source
Primary effectiveness outcome			
Exclusive MOM feeding at discharge	Exclusive MOM feeding at discharge (yes/no; summarized as proportion)	Patient-specific timepoints at NICU discharge	Medical and nursing records
Secondary effectiveness outcomes			
Feeding status at discharge	Feeding status at discharge, categorized as exclusive MOM, mixed feeding (MOM + formula), or exclusive formula	Patient-specific timepoints at NICU discharge	Medical and nursing records
Occurrence of complications	Occurrence of at least one major complication during NICU stay (yes/no)	Patient-specific timepoints at NICU discharge	Medical and nursing records
Length of stay	Mean length of NICU stay	Patient-specific timepoints at NICU discharge	Medical and nursing records
Receipt of HDM	Any exposure to HDM during NICU stay (yes/no)	Patient-specific timepoints at NICU discharge	Medical and nursing records
Pumping or breastfeeding behaviour	Initiation of lactation (yes/no)	4 weeks p.p.	Survey of the mothers of enrolled VLBW infants
Breastfeeding/pumping self-efficacy	Adapted version of the Breastfeeding Self-Efficacy Scale-Short form [42]	4 weeks p.p.	Survey of the mothers of enrolled VLBW infants
Willingness to donate milk	Self-developed item; binary elicitation of attitude toward BM donation	4 weeks p.p.	Survey of the mothers of enrolled VLBW infants
Willingness to accept HDM	Self-developed item; binary elicitation of attitude toward accepting HM donation	4 weeks p.p.	Survey of the mothers of enrolled VLBW infants
Amount of milk pumped	Amount of milk expressed by day 14 postpartum (ml)	4 weeks p.p.	Survey of the mothers of enrolled VLBW infants
Implementation outcomes			
Acceptability	Acceptability of Intervention Measure (AIM) [43]	NICU-specific timepoint, at the beginning of the intervention phase	NICU staff survey
Appropriateness	Intervention Appropriateness Measure (IAM) [43]	NICU-specific timepoint, at the beginning of the intervention phase	NICU staff survey
Feasibility	Feasibility of Intervention Measure (FIM) [43]	NICU-specific timepoint, at the beginning of the intervention phase	NICU staff survey
Intervention fidelity	Adherence, dose delivered, service quality, responsiveness [45]	Staff: at the end of the intervention phase; mothers: 8 weeks p.p.	NICU staff survey, survey of the mothers of enrolled VLBW infants
Implementation fidelity	Adherence, dose delivered, service quality, responsiveness [44,79]	At the end of the intervention phase	NICU staff survey, survey of the mothers of enrolled VLBW infants
Sustainability	NoMAD [46], PRESS [47], SMSS [48]	NICU-specific timepoint, at the beginning and end of the intervention phase	NICU staff survey
Coverage	Reach [35]: number of participating NICUs, infants, and mothers; Comparison with intended population	At the end of the study	Data extracted from recruitment statistics

*MOM* mother’s own milk, *NICU* neonatal intensive care unit, *p.p*. postpartum, *BM* breast milk,* HM* human milk

Consistent with a hybrid type 1 study, which prioritizes effectiveness evaluation, the study additionally included a descriptive assessment of perceptual and behavioural implementation outcomes [33–35]. The hybrid type 1 approach was selected because the primary research question was intended to concern intervention effectiveness on a clinical outcome (exclusive MOM feeding at discharge), with the trial powered accordingly on this patient-level endpoint. Implementation strategies were deployed to support intervention delivery but were not subject to controlled comparison or randomized evaluation.

The study was conducted from April 2022 to March 2024 with a subsequent two-month follow-up. No major external disruptions (e.g., policy changes, pandemic-related restrictions) were identified that systematically affected study conduct. The study was registered prospectively in the German Clinical Trials Register on 6 May 2021 (DRKS00025058; https://drks.de/search/en/trial/DRKS00025058) and approved by the Ethics Committee of the University of Cologne (21-1506) and local ethics committees of participating NICUs. The results are reported according to the Consolidated Standards of Reporting Trials (CONSORT 2025, CONSORT SW-CRT) Statements [36, 37] and the Standards for Reporting Implementation Studies (StaRI) guidelines [38] (Additional file 1). Detailed information regarding the study protocol of the trial [32] and the development of the intervention [29] can be found elsewhere. The statistical analysis plan is not publicly available. It can be obtained from the corresponding author on reasonable request.

### Setting and participants

Fifteen German Level I NICUs (highest German neonatal care level [31]) were recruited via professional networks, selected for diversity in size and organizational culture. Eligibility criteria required openness to HDM and lactation promotion. The original protocol included both Level I and Level II NICUs (German Level II NICUs provide intermediate neonatal care for less critically ill or more mature preterm infants over 1,250 g [31]). However, only Level I NICUs could be recruited, which may limit generalizability to other settings. VLBW infants (< 1,500 g; ICD-10-GM: P07.00, P07.01, P07.02, P07.10, P07.11) were enrolled consecutively. Exclusion criteria were lethal malformations, palliative care, and external transfers. To reduce spill-over between study phases, infants born after the 15th day of the last month of a cluster’s control phase (and their mothers) were not included. Mothers (age ≥ 18 years) provided written consent for surveys and interviews. Healthcare professionals participated via anonymous voluntary surveys: key informants completed implementation process surveys; all NICU staff were invited to implementation outcome surveys.

### Randomization and blinding

The 15 NICUs were randomized to three sequences (sequence allocation ratio 1:1:1, 5 NICUs per sequence) by an independent statistician (computer-generated random allocation) after recruitment completion. Sites were informed of sequence allocation midway through the first control period to minimize contamination.

Blinding of participants and healthcare providers was infeasible given the intervention nature. To reduce analysis bias, the statistician remained blinded during data preparation and finalization of the statistical analysis plan. However, complete blinding during model estimation was not feasible, as cluster IDs and temporal sequence information revealed allocation. To mitigate bias, the analysis models for the outcomes reported here were specified in the statistical analysis plan (final version, 25 October 2024) before model estimation began.

### Intervention and implementation

The Neo-MILK intervention comprised five core components adaptable to local contexts [32]: (1) delivery of structured prepartal and postpartal lactation counselling with evidence-based protocols; (2) question prompt sheet for mothers/parents for prepartal consultation preparation; (3) multilingual educational materials; (4) customizable web application for pumping documentation, and reminders; and (5) on-site HDM banking with standardized protocols (see Additional file 2 [39, 40]). Control groups received standard NICU care, which may have included lactation support by healthcare professionals or HDM access.

Implementation strategies included video-based training for NICU staff, structured guidelines and a checklist for the prepartal consultation, commitment nudges for medical staff, and support for HDMB establishment. All strategies were voluntary and adaptable to local contexts [29, 32] (specified according to Proctor et al. [40] in Additional file 2). They were based on evidence from the literature and two mixed-methods surveys of mothers and pre-existing HDMBs in Germany conducted during the developmental phase of the project [29, 41]. Furthermore, two quantitative surveys of NICU managers at the medical and nursing levels were conducted [29, 41]. Patients and the public were not involved in the design, conduct or reporting of the trial. Mothers and parents of preterm infants contributed to the development of the intervention components and to the piloting of the survey and interview instruments.

### Outcomes

The primary outcome was the individual-level feeding status at discharge, operationalized as the proportion of VLBW infants receiving exclusively MOM on the day of NICU discharge (extracted from medical and nursing records). Feeding categories were defined by source. Exclusive MOM denotes mother’s own milk with no formula or HDM supplementation. Multi-nutrient fortifiers (human milk–based or cow’s milk–based), which were used with both MOM and HDM, were not recorded as a separate category in the case report form and therefore do not affect this classification. Consistent with the trial registration, which defines the primary endpoint at transfer or discharge from the NICU, transfer to a lower level of care was treated as discharge from the NICU.

Secondary outcomes included feeding status at NICU discharge, categorized as exclusive MOM, mixed feeding (MOM and formula), or exclusive formula; occurrence of major complications (necrotizing enterocolitis, bronchopulmonary dysplasia, sepsis); length of hospital stay; and HDM exposure during NICU stay (see Table 1). Maternal outcomes from surveys at 4 and 8 weeks postpartum (p. p.) included breastfeeding/pumping behaviour, breastfeeding/pumping self-efficacy [42], willingness to donate milk, milk volume at day 14 p. p., and intervention component assessments. The outcomes reported here were specified in the study protocol and in the statistical analysis plan (final version, 25 October 2024), which was finalised before the analyses began. Major complications were recorded as prespecified secondary clinical outcomes from medical and nursing records. No separate systematic surveillance of adverse events was conducted.

Implementation outcomes assessed perceived acceptability, feasibility and appropriateness [43], coverage [35], fidelity [44, 45], and sustainability [46–48] via validated surveys of staff and mothers (see Table 1). Sustainability was assessed with three validated instruments measuring distinct constructs: the Normalization MeAsure Development questionnaire (NoMAD), which operationalises normalization of an intervention on the basis of Normalization Process Theory [49] and for which we used the validated German version (G-NoMAD) [46]; the Provider Report of Sustainment Scale (PRESS), capturing provider-reported sustainment of the inner context [47]; and the Sustainment Measurement System Scale (SMSS), capturing sustainment of the programme [48]. Cost outcomes included intervention costs (time-driven activity-based costing [50]), out-of-pocket expenses, and budget impact analysis from a health insurance perspective. Detailed methodology is provided in the Additional file 3 [50–57].

The trial was prospectively registered (DRKS00025058, 6 May 2021). The registered outcome constructs were operationalised in the published study protocol [32] and in the statistical analysis plan (final version, 25 October 2024). No outcome construct was added, removed or redefined after trial commencement. Implementation and economic outcomes form part of the hybrid type 1 design. They were specified in the published study protocol, are reported descriptively, and were not tested confirmatorily.

### Sample size

Assuming a baseline 30% of exclusive MOM feeding and a 1.7-fold increase (α = 0.05, β = 0.20, intra-cluster correlation coefficient [ICC] = 0.05), we calculated the sample size for 15 clusters across three sequences with four measurement periods (6-month intervals). This yielded 960 required VLBW infants. Based on national averages [58], twelve NICUs were needed. We recruited 15 to account for dropout and admission variability. No interim analyses were performed and no stopping guidelines were applied.

### Statistical analysis

All analyses followed intention-to-treat (ITT) principles (α = 0.05). The primary analysis used generalized linear mixed models with random effects for NICUs and fixed effects for time periods adjusting for secular trends [59]. Implementation and cost outcomes were analyzed descriptively. In addition, implementation outcomes were analyzed without preset thresholds, as this was a hybrid type 1 trial.

Secondary analyses included sensitivity analyses adjusting for baseline infant characteristics, and prespecified subgroup analyses by birth weight category (< 1,000 g vs. ≥1,000 g) and illness severity assessed using the Clinical Risk Index for Babies II (CRIB-II) score (range 0–27, with higher scores indicating greater illness severity) [60]. We used CRIB-II (≥ 11 vs. <11) to distinguish higher versus lower illness severity, consistent with risk stratification thresholds used in previous mortality prediction studies [61]. Maternal subgroups were defined by pumped milk volume at 14 days p.p. (< 500 ml vs. ≥500 ml). This threshold reflects failure to achieve ‘coming to volume’, defined as a daily pumped MOM volume of ≥ 500 ml by 14 days p.p., which has been identified as the strongest predictor of continued MOM feeding to NICU discharge in VLBW infants [62]. The ICC was estimated from the data to inform future trial planning. Secular trends were assessed by examining outcomes in control periods across calendar time.

All analyses used available data without imputation. The primary analysis included all 1,627 enrolled VLBW infants. Secondary infant-level analyses used available outcome data. The prespecified sensitivity analysis adjusting for sex and congenital malformations included 1,595 infants. Thirty-two cases (2.0%) were excluded due to missing covariate data on sex or malformation status. Maternal survey outcomes and implementation outcomes were analysed at each timepoint. Non-response was not adjusted for in any analysis. All analyses used R version 4.3.3.

During the preparation and revision of this manuscript, the authors used an AI-based assistant (Claude, Anthropic), under the authors’ direction, to support language editing and restructuring of text passages. The tool was not used to generate, analyse, or interpret study data, to design the study or the analyses, or to create any images. All AI-assisted output was critically reviewed, edited, and verified by the authors, who take full responsibility for the content, accuracy, and integrity of the manuscript.

## Results

### Descriptives

**Fig. 1:**
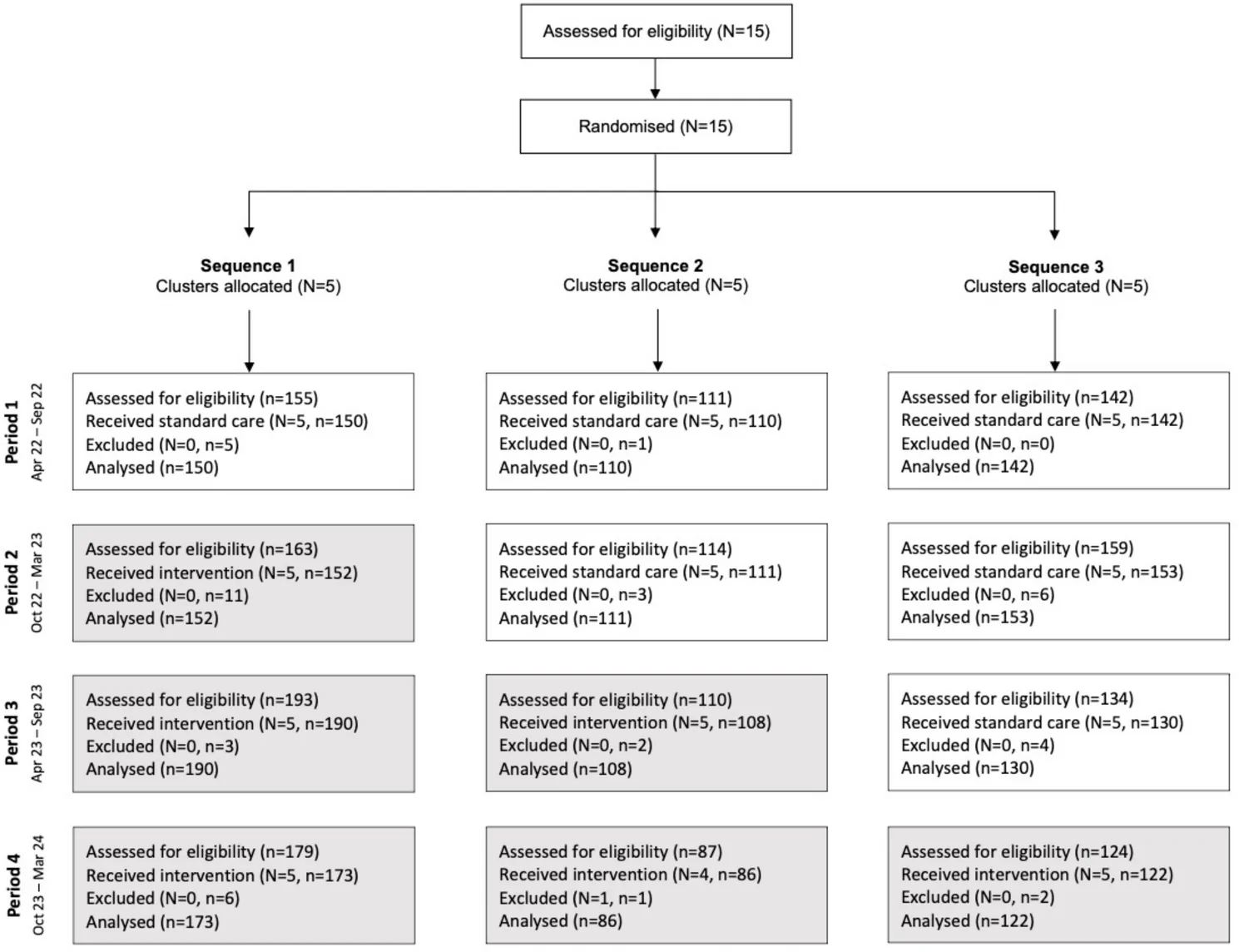
CONSORT flowchart for a stepped-wedge cluster-randomized trial by allocated sequence and period. Note: Gray boxes show intervention phases; white boxes indicate control phases. Reasons for exclusion according to the study protocol: lethal malformation, palliative care, external transfer

Of the 15 participating NICUs, 14 completed the trial (one dropped out in period 4 as a result of a reassessment of study participation by the clinic’s management). Among the 1,627 VLBW infants included, 831 were in intervention periods and 796 in control periods (Fig. 1).

Baseline characteristics of the included VLBW infants showed that the groups were well balanced (Table 2 and Additional file 4). Overall, 49% of infants were female, two thirds were from singleton pregnancies, and 11% had major congenital malformations. Nearly one third (30%) of enrolled infants had a birth weight between 1,250 g and 1,499 g, while 5% of infants weighed less than 500 g at birth. The mean CRIB score was 3.91 (*SD* 3.96), indicating moderate illness severity across the cohort. Most infants (90%) were born by caesarean section.

**Table 2:** Baseline characteristics pooled across all clusters and time periods

	Control	Intervention	Total	SMD
Infants	796 (100%)	831 (100%)	1,627 (100%)	
Female	368 (46.41%)	420 (50.79%)	788 (48.64%)	0.09
Multiple birth	269 (33.79%)	287 (34.54%)	556 (34.17%)	0.02
Malformations	78 (9.94%)	105 (12.73%)	183 (11.37%)	0.09
CRIB-Score, *M* (*SD*)	3.98 (3.99)	3.84 (3.93)	3.91 (3.96)	0.03
**Mode of delivery**				
Spontaneous birth	71 (8.92%)	74 (8.90%)	145 (8.91%)	0.00
Caesarean	633 (79.52%)	686 (82.55%)	1,319 (81.07%)	0.08
Emergency caesarean	82 (10.30%)	66 (7.94%)	148 (9.10%)	0.08
**Birth weight**				
< 500 g	42 (5.28%)	43 (5.17%)	85 (5.22%)	0.00
500–749 g	156 (19.60%)	142 (17.09%)	298 (18.32%)	0.06
750–999 g	185 (23.24%)	185 (22.26%)	370 (22.74%)	0.02
1,000–1,249 g	190 (23.87%)	197 (23.71%)	387 (23.79%)	0.00
1,250–1,499 g	221 (27.76%)	260 (31.29%)	481 (29.56%)	0.08
**Reason for preterm birth**				
Contractions	267 (33.54%)	255 (30.69%)	522 (32.08%)	0.06
Clinical chorioamnionitis	95 (11.96%)	84 (10.11%)	179 (11.02%)	0.06
Preeclampsia	91 (11.43%)	128 (15.40%)	219 (13.46%)	0.12
HELLP	29 (3.64%)	43 (5.18%)	72 (4.43%)	0.07
Pathologic CTG	153 (19.22%)	198 (23.83%)	351 (21.57%)	0.11
IUGR	159 (19.97%)	182 (21.90%)	341 (20.96%)	0.05
Anhydramnios	9 (1.13%)	11 (1.32%)	20 (1.23%)	0.02
Rupture of the membranes	67 (8.42%)	51 (6.14%)	118 (7.25%)	0.09
Placenta abruption	44 (5.53%)	48 (5.78%)	92 (5.65%)	0.01

*CRIB* Clinical Risk Index for Babies, *HELLP* Hemolysis Elevated Liver enzymes Low Platelet count, *CTG* Cardiotocography,* IUGR* Intrauterine Growth Restriction, Clinical chorioamnionitis was recorded clinically at birth (including suspected cases) as an indication for preterm birth, *SMD* standardised mean difference between groups, SMD < 0.10: negligible, 0.10 ≤ SMD < 0.20: small. Congenital malformations were documented at birth and classified according to ICD-10-GM (Chapter XVII, Q00-Q99). Percentages are based on available data.

Survey responses were received from 307 mothers, of whom 290 could be allocated to a study group (163 control, 127 intervention). This corresponds to an overall response rate of 23.2% (307/1,326 eligible mothers), with group-specific rates of 24.9% (163/655) in the control and 18.9% (127/671) in the intervention condition. Characteristics were balanced across groups (Additional file 4).

### Primary effectiveness outcome

The intervention significantly increased exclusive MOM feeding at NICU discharge by 11 percentage points (95% confidence interval [CI] 0.03 to 0.19, *p* = 0.007) compared to the control condition (Table 3). The baseline rate of exclusive MOM feeding in the control condition was 43%. No significant time trends were observed, and ICC was low (0.01). Subgroup analyses showed positive but non-significant effects in extremely low birth weight infants (< 1,000 g; *n* = 753; coefficient 0.04, 95% CI − 0.08 to 0.17, *p* = 0.48) and negative and non-significant effects in high-risk infants (CRIB ≥ 11; *n* = 112; coefficient − 0.08, 95% CI − 0.36 to 0.21, *p* = 0.60). The sensitivity analysis adjusting for sex and congenital anomalies was consistent with the primary analysis (coefficient 0.12, 95% CI 0.04 to 0.20, *p* = 0.003). The intervention effect remained significant, with severe congenital anomalies showing a negative association with exclusive MOM feeding at NICU discharge (coefficient − 0.10, 95% CI − 0.18 to − 0.03, *p* = 0.009). Sex was not significantly associated with the outcome. Site-specific intervention effects on the primary outcome are shown in Additional file 5. Effects varied across the 15 participating NICUs. Sites that had established an HDMB did not show systematically larger effects.

**Table 3:**
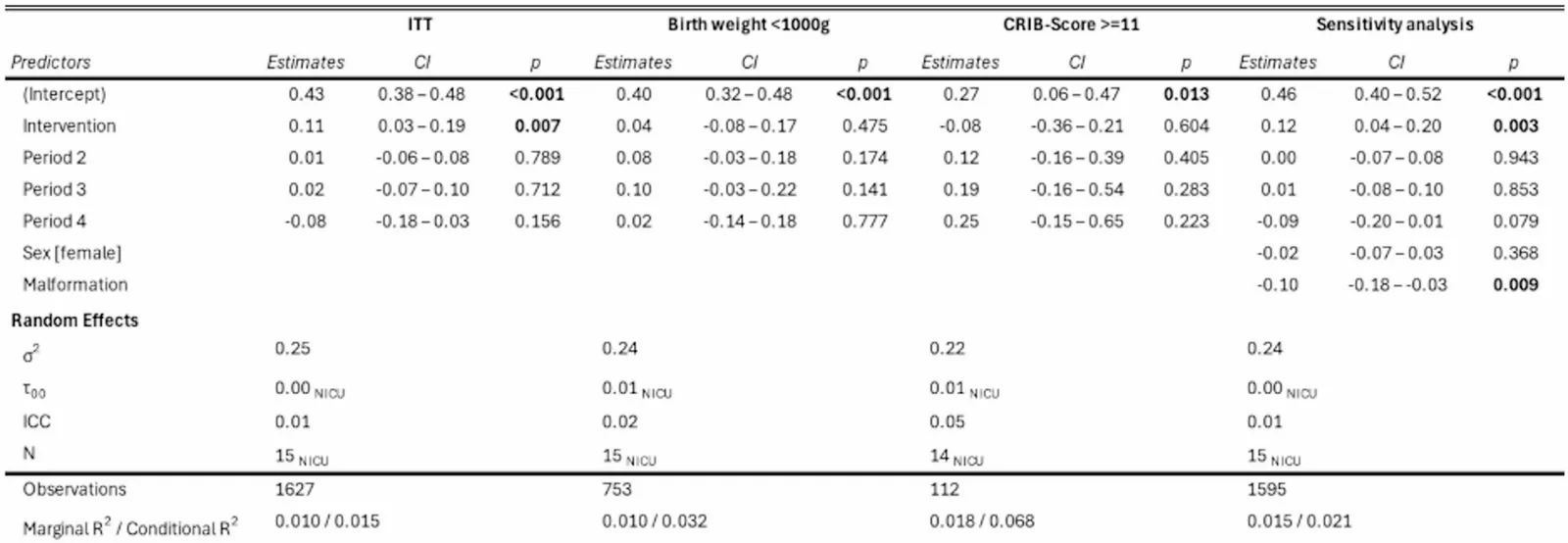
Regression models for the primary effectiveness outcome (exclusive MOM feeding at discharge)

*ITT* Intention to treat, *CRIB* Clinical Risk Index for Babies, *ICC* intra-cluster correlation coefficient

### Secondary outcomes

Beyond the primary outcome, we examined a range of secondary effectiveness outcomes at the infant and maternal level, including feeding patterns, major complications, HDM receipt, length of NICU stay, lactation initiation, willingness to donate and accept HDM, breastfeeding self-efficacy, and milk volume at 14 days postpartum. Secondary effectiveness outcomes are presented with 95% confidence intervals only. In line with our statistical analysis plan, no adjustment for multiple testing was made.

#### Infant outcomes

**Fig. 2:**
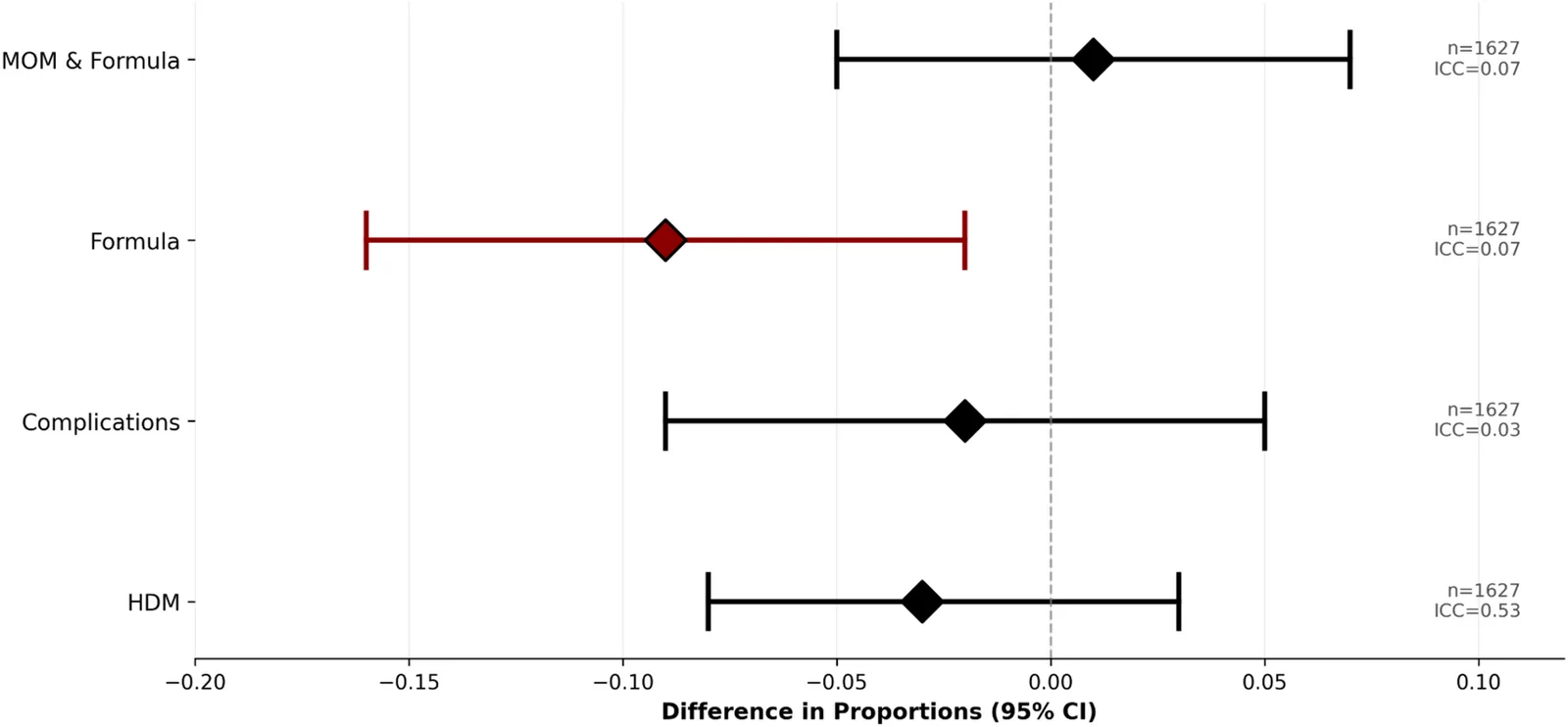
Intervention effects on secondary effectiveness outcomes at infant level

The intervention showed differential effects across feeding categories (Fig. 2 and Additional file 5). For mixed feeding (MOM and formula), the intervention was associated with minimal change (coefficient 0.01, 95% CI − 0.05 to 0.07), with a baseline rate of 16% (95% CI 11 to 22%). In contrast, exclusive formula feeding decreased in the intervention group (coefficient − 0.09, 95% CI − 0.16 to − 0.02) from a baseline rate of 27% (95% CI 20 to 33%). These complementary findings align with the increased exclusive MOM feeding observed in the primary outcome. The occurrence of major complications showed a minimal difference between groups (coefficient − 0.02, 95% CI − 0.09 to 0.05). The baseline complication rate was 24% (95% CI 18 to 29%), with no meaningful variation across study periods. Receipt of (external) HDM during NICU stay was similar between groups (coefficient − 0.03, 95% CI − 0.08 to 0.03; baseline 25%), with high ICC (0.53) reflecting variation in HDM availability and utilization practices across participating NICUs. Notably, the fourth study period showed an increase in HDM establishment and use (coefficient 0.09, 95% CI 0.02 to 0.16), suggesting evolving practices over the study duration.

**Fig. 3:**
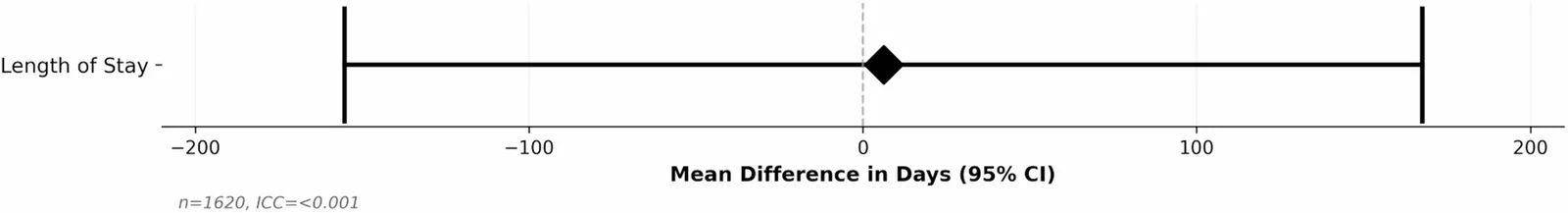
Intervention effect on length of stay

Length of NICU stay showed no meaningful difference between groups (coefficient 6.20 days, 95% CI − 155.25 to 167.66), with a baseline mean of 49.10 days (Fig. 3 and Additional file 5). The extremely wide confidence intervals and large random effect variance (σ² = 1,266,927) indicated substantial heterogeneity in length of stay, probably reflecting the diverse clinical complexity of the study population. This conclusion remained unchanged after additional adjustment for gestational age (Additional file 5).

#### Maternal outcomes (Additional file 5)

Maternal outcomes showed minimal intervention effects. Lactation initiation remained consistently high across both groups (baseline 94%, no change). Willingness to donate (baseline 92%, coefficient 0.03, 95% CI 0.01 to 0.04) and willingness to accept HDM (baseline 68%, coefficient − 0.10, 95% CI − 0.12 to − 0.09) were slightly different between groups, with notably lower acceptance than donation rates. Breastfeeding Self-Efficacy Scale scores did not differ significantly between groups (coefficient 2.41, 95% CI − 3.94 to 8.75; baseline 55.34). The proportion of mothers achieving ≥ 500 ml pumped milk at 14 days p.p. (self-reported) showed no difference between groups (OR 0.91, 95% CI 0.44 to 1.88; baseline OR 0.63). In an exploratory subgroup analysis (Additional file 5), mothers of infants with a (mother-reported) birth weight < 1,000 g reached this threshold less often than mothers of infants ≥ 1,000 g (51/144, 35.4% [95% CI 27.6 to 43.2] vs. 52/123, 42.3% [95% CI 33.5 to 51.0]). After adjustment for study period and intervention group, this descriptive difference was not confirmed and was directionally reversed (adjusted OR for < 1,000 g: 1.30, 95% CI 0.77 to 2.18, *p* = 0.321).

### Implementation outcomes

Across all 15 Level I NICUs, the intervention reached 831 of 856 VLBW infants assessed for eligibility during the intervention phases (97.1%) and their 671 mothers. The 25 infants not enrolled received parts of the intervention but were not included owing to lethal malformation, palliative care, or external transfer. The participating NICUs represented diverse organizational contexts comparable to German Level I NICUs nationally (Additional file 4). Of the 259 responses received from NICU staff, 67.2% (*n* = 174) reported familiarity with the intervention content. A pre-implementation assessment of these staff indicated high levels of acceptability (83–92%), appropriateness (83–87%), and feasibility (70–85%). Post-implementation assessment was reported by 66 healthcare professionals, limiting interpretation of these measures. Fidelity of core intervention components was reported by them as strong, with prepartal counselling delivered according to protocol in 71% of cases and a mean self-reported reach of 8.4 out of 10 eligible mothers per site, based on staff estimates in the NICU staff survey. Information materials achieved near-universal distribution (8.4/10 parents), with high maternal satisfaction (> 95% understanding, 96% feeling respected). HDMBs were locally established in 40% of participating NICUs. No participating NICU operated a HDMB before the study.

**Fig. 4:**
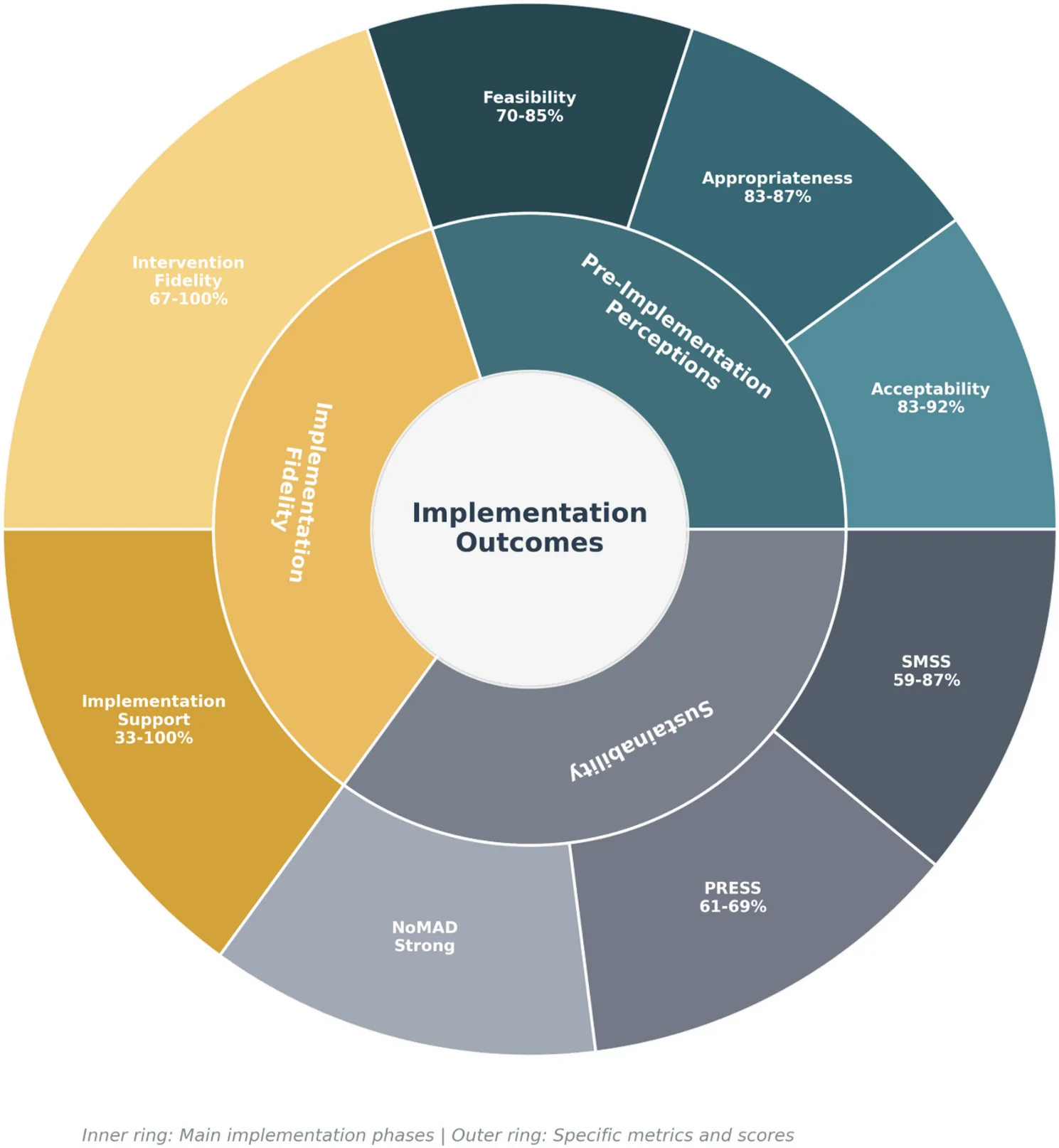
Summary of implementation outcomes

Implementation strategies showed variable uptake (51% of staff receiving training invitations, material utilization ranging from 30% [pocket guides] to 67% [handbook]). Furthermore, sustainability indicators were promising: NoMAD showed successful normalization (75–95% across domains), while 61% of staff reported integration of intervention components into routine care (PRESS) and 87% reported sustainment indicators consistent with alignment to organizational values (SMSS). Figure 4 summarizes the results in a sunburst diagram showing implementation across the three phases of pre-implementation preparation, implementation (fidelity), and maintenance (sustainability). The outer ring displays specific metrics within each phase, where percentages represent the proportion of staff rating components positively for pre-implementation domains, self-reported adoption rates and adherence levels for fidelity components, and sustained application indicators. Detailed implementation metrics are provided in Additional file 4.

### Economic evaluation

Mean intervention costs were €649 per infant. Budget impact analysis for the German target population (3,909 infants < 1,500 g over 6 months) projected net savings of €810,176 nationally (base-case scenario). Economic viability was found to be highly sensitive to implementation quality, however, with savings ranging from €2.8 million (best case: 77% exclusive MOM feeding) to costs of €1.9 million (worst case: 33% exclusive MOM feeding). Out-of-pocket expenses were similar between groups (intervention €146 vs. control €156). The full economic analyses are provided in Additional file 3.

## Discussion

### Principal findings

In this stepped-wedge cluster-randomized trial, structured lactation support combined with HDMBs increased exclusive MOM feeding rates at NICU discharge among VLBW infants. The 11-percentage-point increase is consistent with international evidence on structured lactation interventions [16, 24] and represents a clinically meaningful improvement achieved across diverse hospital settings. The intervention’s effectiveness was observed in the context of its multi-component design, which included prepartal counselling and comprehensive postpartal support. Ward et al. (2012) identified (prepartal) counselling and education as critical for early lactation success [19], and our intervention systematically integrated these elements from the prepartal period through NICU discharge.

While Arslanoglu et al. (2013) showed that HDMB availability is associated with higher exclusive breastfeeding at NICU discharge [24], our results show improvements in MOM feeding also in a setting in which HDMB infrastructure was not universally available (only 40% of our sites had established HDMBs). This may indicate that lactation support is the more influential component, with HDMBs providing complementary infrastructure. Because the components were not randomised separately, their individual contributions cannot be disentangled.

Williams et al. (2016) emphasized that implementing HDMB does not obviate the need for maternal lactation support. Both strategies work synergistically [17]. Our findings are consistent with this pattern: lactation support was associated with higher exclusive MOM feeding rates, while HDM availability may provide a safety net when maternal supply is temporarily insufficient, together potentially reducing formula exposure. This integrated approach was associated with improved exclusive MOM feeding even when HDMBs were not universally established, in the context of comprehensive lactation education, staff training, and proactive lactation management. Sites that successfully established HDMBs reported that HDM availability reinforced staff commitment to HM feeding and reduced provider anxiety about nutritional adequacy when MOM was limited. While comprehensive lactation support may contribute to improvements on its own, HDM banking as an integrated component may strengthen the system’s resilience and support a broader shift toward prioritizing HM in NICU care. These interpretations are hypotheses generated by the trial rather than tested effects. The lower willingness to accept HDM in the intervention group may reflect the intervention’s strengthened focus on, and greater success in establishing, MOM provision. As mothers more often succeeded in providing their own milk, their perceived need for donor milk may have decreased, and with it their stated willingness to accept it.

No increase in neonatal complications or length of NICU stay was observed, providing no indication of harm from intensive lactation promotion in medically fragile neonates. These were prespecified secondary clinical outcomes rather than systematically collected harms. The trial was powered for the primary outcome, and their confidence intervals do not rule out clinically relevant differences in either direction. While lactation support is generally beneficial and safe, some literature highlights potential risks in vulnerable NICU populations. For instance, rigid policies may unintentionally pressure mothers, leading to guilt or elevated stress when breastfeeding goals are not met [63], and promotional messaging may be experienced as overly moralized by mothers facing difficulties [64]. However, systematic reviews of family-centered care interventions in NICUs have reported improved infant feeding and parent mental health without adverse outcomes, suggesting that these concerns may be mitigated when lactation support is delivered within a respectful, family-focused framework [65]. Our study achieved improvement in MOM feeding rates in the context of a supportive approach intended to avoid pressure. This is particularly important given that maternal stress has been associated with reduced milk production [66, 67].

Subgroup analyses revealed positive but smaller and non-significant effects in the most immature infants (< 1,000 g) and a negative but non-significant estimate in high-risk infants (CRIB ≥ 11). These results may be due to the smaller sample size, or they may suggest that these populations face greater challenges that potentially require more intensive support. Maternal stress may be a critical mediator [66, 67], as stress levels increase with infant prematurity and medical complexity. Stress is associated with increased cortisol, adrenaline, dopamine, and other mediators of stress, as well as inhibition of prolactin and oxytocin, hormones central to milk production [66, 67]. Early lactation initiation, a key factor for successful lactation after preterm birth [68, 69], may be particularly affected. Enhanced strategies for mothers to cope with emotional stress [70, 71] may be required to improve outcomes in these highest-risk infants. The smallest and sickest infants also experience the longest NICU stays, during which mothers typically undergo the longest period of exclusive expressing before direct breastfeeding becomes feasible. In an anonymous nationwide survey conducted in preparation for our study, exclusive pumping was associated with earlier cessation of MOM feeding after discharge, whereas an adequate early milk volume (> 500 ml/day by day 14) was protective during the NICU stay [72]. An early milk volume ≥ 500 ml/day at day 14 p.p. is likewise an established predictor of continued MOM feeding to discharge [62]. This may contribute to the attenuated intervention effect observed in the lowest-birth-weight subgroup.

We encountered challenges reflecting complex healthcare implementation [73]. Among NICU staff, 51% perceived training invitations, with uptake rates of 60% for the short version (2 h) and 11% for the extended version (9 h). Material utilization in daily practice ranged from 30% (pocket guides) to 67% (handbook). These findings emphasize the importance of active implementation strategies including protected training time, leadership buy-in, and on-unit champions to overcome competing priorities and resource constraints. The pragmatic nature of our trial meant that implementation fidelity varied across sites, reflecting real-world conditions where resource availability, regulatory environments, and organizational cultures differ. Consistent with this, intervention effects on the primary outcome varied across sites, with no systematic advantage for those that had established an HDMB. This variability is informative for future implementation efforts and highlights the need for continued support beyond initial rollout, including ongoing training, audit and feedback, and organizational culture change to sustain practice improvements. Among the intervention components, establishing HDMB was particularly challenging, with only six of the 15 sites successfully establishing operational HDMBs, reflecting the substantial regulatory, infrastructural, and resource requirements. Nevertheless, sustainability indicators were promising and indicated the potential for long-term adoption with adequate support.

The economic evaluation showed potential for intervention sustainability and healthcare system benefits. Despite costs of €649 per infant, the budget impact analysis projected net savings of €810,176 nationally over 6 months in the base-case scenario. These results are model-based estimates from a deterministic budget impact analysis under stated assumptions. As no probabilistic sensitivity analysis was performed, decision uncertainty is characterised through deterministic best-case and worst-case scenarios rather than probabilistically, and the estimates should be interpreted with corresponding caution. Out-of-pocket costs for families were modest and similar between groups (intervention €146 vs. control €156), dominated by breast pump expenses, suggesting that the intervention does not create financial barriers. Economic viability was found to be highly sensitive to implementation quality, however, with savings ranging from €2.8 million (best case: 77% exclusive MOM feeding) to costs of €1.9 million (worst case: 33%). The true economic benefit is probably greater than the short-term estimate, given the 6-month analysis period. Even if such programs appear cost-neutral in the short run, they may yield strong returns on investment when considering lifelong health and developmental advantages for VLBW infants. These institutional estimates do not capture the opportunity cost of parental time. Establishing and maintaining MOM provision requires intensive, largely unpaid maternal labour (e.g., frequent expressing), so a reduction in institutional formula expenditure may in part shift costs onto families as time and effort. This opportunity cost is not reflected in the payer-perspective budget impact model and should be weighed alongside the institutional savings reported here [74].

### Strengths and limitations

The stepped-wedge design provided efficient evaluation across fewer clusters than parallel designs [75]. Having all NICUs receive the intervention increased statistical power and enhanced acceptability to sites. We also observed a lower-than-expected ICC of 0.01 (vs. the 0.05 assumed), suggesting that feeding outcomes were more independent across sites than anticipated.

However, limitations merit consideration. The stepped-wedge design with four time periods limits the power to detect complex time-varying effects and intervention-period interactions. We did not observe significant secular trends, but longer observation periods might reveal more complex temporal patterns. Furthermore, the study was conducted exclusively in Level I NICUs, potentially limiting generalizability to lower-level neonatal units with different resources and patient populations. The generalizability of the findings may also be limited by the above-average percentage of C-sections in the mode of delivery of the mothers in the study sample. This may indicate differences in practice style of the studied NICUs compared to the international context [76]. In addition, maternal outcome data were collected from a subset of participants, introducing potential selection bias toward more educated and motivated mothers. Moreover, the analysis followed ITT principles based on randomized sequences rather than actual implementation dates. While this preserves the randomization, it may underestimate the true intervention effect, given implementation delays in some clusters. Finally, the intervention’s multi-component nature makes it difficult to determine the relative contribution of individual components to the observed effects.

### Implications for policy, practice, and future research

This trial supports WHO 2022 recommendations emphasizing structured lactation support as core neonatal care [77], and our findings are consistent with systematic programmes contributing to improvements across diverse settings. However, passive dissemination of guidelines is insufficient; dedicated resources (lactation consultants, pumping equipment, HDMB infrastructure where feasible), staff training, and ongoing quality improvement are essential for sustained success.

Future research priorities include long-term follow-up to assess whether improvements in exclusive MOM feeding translate into sustained breastfeeding duration after discharge and longer-term neurodevelopmental outcomes. Additionally, refining interventions for highest-risk infants (< 1,000 g) and developing innovative delivery mechanisms (e.g., tele-lactation, digital support extending beyond discharge) represent important research directions. Given the regulatory challenges observed, research on HDMB network models (centralized vs. distributed) would inform resource allocation decisions.

## Conclusions

The Neo-MILK trial shows that providing structured lactation support alongside HDMB establishment improved exclusive MOM feeding among VLBW infants at NICU discharge by 11 percentage points across 15 diverse sites, which suggests transferability beyond single-centre settings. Despite implementation challenges, no increase in complications or length of stay was observed, and the intervention may be cost-saving. At the same time, the trial underscores the complexity of embedding such interventions into everyday practice, emphasizing that sustained success requires dedicated resources, ongoing quality improvement, and organizational commitment to fostering a positive MOM feeding culture.

Taken together, our results suggest that future research and implementation efforts should prioritize three areas: (1) refining and tailoring interventions for the highest-risk infants (< 1,000 g); (2) developing innovative strategies to integrate and sustain lactation support, potentially through digital health, tele-lactation services, or other mechanisms that extend support beyond discharge; and (3) rigorously assessing long-term health outcomes to understand the sustained impact of improved early nutrition.

## Supplementary Information

Below is the link to the electronic supplementary material.

Supplementary Material 1: Additional file 1: Reporting checklists

Supplementary Material 2: Additional file 2: Description of the intervention and implementation strategies

Supplementary Material 3: Additional file 3: Health economic evaluation

Supplementary Material 4: Additional file 4: Implementation metrics

Supplementary Material 5: Additional file 5: Secondary effectiveness outcomes

Supplementary Material 6: Additional file 6: Variable codebook

## Data Availability

The data supporting the findings of this study are subject to controlled access. They are not made generally or publicly available. Under the study’s approved data protection concept and the conditions of the ethics approval granted by the Ethics Committee of the University of Cologne (reference 21-1506), the CRF data were collected under a waiver of informed consent. The ethics committee granted this waiver, which was essential to achieve complete enrolment of all eligible very-low-birth-weight infants (a full cohort census) and thereby to avoid consent-related selection bias, on the condition that the data are not made generally available; accordingly, the patient-level CRF data are not published openly and are not deposited in any public or external repository. This restriction does not preclude controlled, case-by-case sharing. A de-identified minimal data set (i.e., the variables underlying the analyses reported in this article) may be released to qualified researchers, on request, for non-commercial academic research and transferred under a data use agreement (DUA). Requests should be directed to the corresponding author, Prof. Dr. Juliane Köberlein-Neu, at Versorgungsforschung@wiwi.uni-wuppertal.de. Requests must include a research proposal specifying the intended analyses together with evidence of approval by an appropriate research ethics committee. Applicants will normally receive a decision within eight weeks of submission of a complete request. The DUA restricts use to the approved purpose, prohibits any attempt to re-identify individuals, prohibits onward transfer or redistribution to third parties, requires secure handling, and requires deletion of the transferred data by the recipient on completion of the approved project. The DUA does not impose authorship as a condition of access. A copy is available from the corresponding author on request. The minimal data set remains available under controlled access until ten years after the end of the project, after which the original data will be securely deleted in accordance with the approved data protection concept. The maternal survey data and the data from the health-professional (staff) surveys cannot be shared, because the consent obtained from these participants does not cover the sharing of their individual data and because confidentiality was assured to the respondents. This restriction applies irrespective of the CRF access arrangements described above. A structured description of the study and the complete variable codebook are openly available in the German Central Health Study Hub (NFDI4Health) [78]. The variable codebook is additionally provided as Additional file 6. The statistical code underlying the analyses reported in this article is subject to the same controlled-access conditions as the minimal data set and is shared together with the data. Aggregated data underlying the main findings are reported within the manuscript and its Additional files.

## Abbreviations

CRIB: Clinical Risk Index for Babies
HDM: Human donor milk
HDMB: Human donor milk bank
HM: Human milk
ICC: Intracluster correlation coefficient
ITT: Intention-to-treat
MOM: Mother’s own milk
NICU: Neonatal intensive care unit
VLBW: Very low birth weight
