# Supplementary material for: Effectiveness of structured lactation support and human donor milk banking in German NICUs: a stepped-wedge cluster-randomized trial
Source: BMC Med. 2026 Sep 11;24:504. doi: 10.1186/s12916-026-05211-1 (PMC13587408; doi:10.1186/s12916-026-05211-1)
Supplement: Supplementary file 1 [file 12916_2026_5211_MOESM1_ESM.docx]

**Effectiveness of Structured Lactation Support and Human Donor Milk Banking in German NICUs: A Stepped-Wedge Cluster Randomized Trial**

**Authors:** Juliane Köberlein-Neu¹, Nicola Gabriela Dymek¹, Viola Zimmer¹, Trutz Bommhardt^1^, Anna Katharina Stirner², Isabella Güldenring^4^, Tim Ohnhäuser³, Daniel Wiesen²^,5^, Till Dresbach^6^, Nadine Scholten³^,4^; the Neo-MILK Consortium

**Affiliations:** ¹Centre for Health Economics and Health Services Research, University of Wuppertal, Wuppertal, Germany; ²Department of Business Administration and Health Care Management, University of Cologne, Cologne, Germany; ³Department of Health Services Research, University of Cologne, Cologne, Germany; ⁴Department for Psychosomatic Medicine and Psychotherapy, Centre for Health Services Research and Communication (CHSR), University Hospital Bonn, Bonn, Germany; ^5^Erasmus School of Health Policy & Management (ESHPM), Erasmus University Rotterdam, Rotterdam, Netherlands; ^6^Department of Neonatology, University Hospital Bonn, Bonn, Germany

**Additional File 2**

**Description of intervention components and their implementation strategies**

Table 1: Components of the Neo-MILK intervention in accordance with TIDieR [39]

| Brief name | **Why?** | **What?** | **Who provided?** | **How?** | **Where?** | **When and how much?** |
| --- | --- | --- | --- | --- | --- | --- |
| Prepartum consultation on lactation and breastfeeding between mothers, i.e. parents, and staff member of the NICU | The prepartum consultation aims to educate, raise awareness and increase knowledge in the area of lactation and breastfeeding in mothers/parents. | The prepartum consultation provides information on significant topics such as (1) bonding and first contact of the mother, i.e. parents with the VLBW infant, (2) the impact of breastfeeding, colostrum and breast milk, (2) lactation in prematurity, (3) expression of breast milk, (4) hygienic handling of expressed breast milk as well as (5) information on NICU accessibility and (6) contact and information for the respective clinic. | The prepartum consultation is provided by a neonatologist working on the respective NICU. | The prepartum consultation should be delivered to mothers, i.e. parents, individually. | The consultations take place in a separate room of the clinic (e.g. patient room, physician office). | The one-time consultation is intended to take place prepartum. In case of an unexpected birth, information should be provided to mothers/parents after the VLBW infant has been admitted to the NICU. |

| Brief name | **Why?** | **What?** | **Who provided?** | **How?** | **Where?** | **When and how much?** |
| --- | --- | --- | --- | --- | --- | --- |
| Lactation and breastfeeding support of mothers | Ongoing support aims to help mothers initiate, establish and maintain lactation, to support the provision of mother's own milk, and to facilitate the transition toward direct breastfeeding, thereby improving human-milk feeding of the VLBW infant. | Individual lactation and breastfeeding support according to manuals content (see implementation strategies), adapted to the situation of mother and infant. | Neonatologists together with other professionals, e.g. parent counsellors, NICU nursing staff, and lactation consultants. | Delivered face-to-face and individually to mothers/parents as part of routine NICU care. | In the NICU | Continuously throughout the infant's NICU stay |

| Brief name | **Why?** | **What?** | **Who provided?** | **How?** | **Where?** | **When and how much?** |
| --- | --- | --- | --- | --- | --- | --- |
| Question prompt sheet (QPS) for the preparation of prepartum consultation on lactation and breastfeeding between mothers, i.e. parents, and staff member of the neonatal intensive care unit (NICU) | The QPS offers example questions on significant topics such as lactation after preterm birth, bonding, and hygienic handling of expressed breast milk, in line with the information provided during the prepartum consultation. The QPS functions as a preparation aid for mothers, or parents, for the prepartum consultation, and a reference resource for later questions or for refreshing previously acquired knowledge from the consultation. | The QPS is provided as a printed booklet. It provides sample questions on the topics addressed in the prepartum consultation.  Also, there is additional space for questions and further notes. Moreover, simplified definitions i.e., explanations of technical terms relevant to prepartum counselling are included.  To prepare for the consultation or follow-up consultations after delivery, mothers, i.e. parents read through the booklet and may add questions. The QPS is then taken to consultations with HCPs to support communication and collect information provided during the consultation using pages provided for notes. It can be reviewed at any time, both between and after consultations. | The QPS is provided by healthcare providers (HCPs) working on the respective NICU (trained nurses, midwives and physicians). | The QPS should be delivered to mothers, i.e. parents, individually. Mothers, i.e. parents administer the QPS individually. | The QPS should be delivered to mothers, i.e. parents, in a separate room of the clinic (e.g. patient room, physician office). | The QPS is intended for a one-time administration before the first (prepartum) consultation.  The same QPS can however be used multiple times by mothers, i.e. parents for follow-up consultations or for self-study to refresh their knowledge. |
|  |  |  |  |  |  |  |
| Brief name | **Why?** | **What?** | **Who provided?** | **How?** | **Where?** | **When and how much?** |
| Flyers on lactation, breastfeeding, and human donor milk | The provision of flyers aims to educate, raise awareness and increase knowledge in the area of lactation, breastfeeding and human donor milk in mothers/parents. | The printed flyers are written in German and provide information on the following nine topics: (1) Neo-MILK app in general, (2) Neo-MILK films within the app, (3) information for fathers and partners, (4) manual let-down, (5) hygienic handling of expressed breast milk, (6) physical contact and bonding/ kangarooing, (7) strategies for solving lactation or breastfeeding related problems, (8) expression of breast milk, and (9) human donor milk.  In addition, four flyers with the topics 4, 5, 7, 8) are available in Russian, Arabic, Turkish, French, and English. | Flyers are provided by HCPs working on the respective NICU. | Flyers should be delivered to mothers, i.e. parents, individually by handing them over. Additionally, the flyers should be displayed by HCPs on the NICU (e.g., in pumping rooms, ward rooms). | Flyers should be handed over and be displayed on the NICU. | Flyers are intended for a one-time administration to mothers/parents but they should be refilled when not anymore available on the NICU.  The flyers can however be used multiple times by mothers, i.e. parents for self-study to refresh their knowledge. |

| Brief name | **Why?** | **What?** | **Who provided?** | **How?** | **Where?** | **When and how much?** |
| --- | --- | --- | --- | --- | --- | --- |
| Posters on lactation, breastfeeding, feeding VLBW infants with mother’s own milk (MOM), and information on the specificity of the respective NICU being a trial site | The purpose of displaying posters is to educate, raise awareness and increase knowledge in the area of lactation, breastfeeding, and feeding VLBW infants with MOM in mothers/parents. Additionally, the posters should provide information on the specificity of the respective NICU being a trial site. | The printed posters are written in German, available in different sizes (DIN-A4 and DIN-A3), and provide information on the following topics: (1) feeding VLBW infants with MOM, (2) breastfeeding positions, (3) effective expression of breast milk, (4) bonding/ kangarooing, (5) DOs on the NICU, (6) Neo-MILK app, (7) specificity of the respective NICU being a trial site, (8) body care, (9) breast milk, (10) breast milk flow chart | Posters are provided by HCPs working on the respective NICU. | Posters should be made accessible to everyone on the NICU, e.g. by hanging them up. | Posters should be opened in the NICU, e.g. in the NICU corridors as well as in the pumping and breastfeeding rooms. | Posters are intended for a one-time administration, but can be extended or repositioned at any time.  The posters can however be used multiple times by mothers, i.e. parents for self-study to refresh their knowledge. |

| Brief name | **Why?** | **What?** | **Who provided?** | **How?** | **Where?** | **When and how much?** |
| --- | --- | --- | --- | --- | --- | --- |
| App-based support on lactation and breastfeeding and to reflect on breast milk production | The provision of app-based support aims to educate, raise awareness and increase knowledge in the area of lactation, breastfeeding and is intended to help mothers reflect on their breast milk production | The app can only be accessed online via **https://app.neo-milk.de/#/welcome**. It is not possible to install the app on a mobile device.  The app contains information and instructions on the topics of skin-to-skin contact, breast milk, breast milk expression and everyday tips. Informational content in film format is also available. Pumping reminders can be set up to document the milk quantity and pumping/breastfeeding times as well as the pumping/breastfeeding duration with the help of a stopwatch. This can be used to create a personalised milk chart. The app also offers the option of creating diary entries, notes and a picture gallery. Information on the study and the intervention is also provided. | Information on the app and the access to it are displayed on printed flyers and posters, which are provided by HCPs working in the respective NICU. | The app can be accesses and used online. There is a QR code on the flyer containing information about the films to make the website easily accessible.  Registration with an email address is required to use all features. | The app can be accessed and used online from home, on the move or from the NICU. | The access and use of the app are voluntary. There is no specified time frame for using the app. |

| Brief name | **Why?** | **What?** | **Who provided?** | **How?** | **Where?** | **When and how much?** |
| --- | --- | --- | --- | --- | --- | --- |
| Human donor milk bank (HDMB) | The establishment or expansion of HDMB will provide the opportunity to access HDM from the first day of life and, if needed, until discharge. | Every VLBW infant, who is in need for HDM will have access until discharge. Furthermore, mothers of VLBW infants and who are potential donors have the opportunity to donate. | The need for HDM and the potential to donate are assessed by HCPs working in the NICU. | Procedures depend on NICU-specific HDMB concepts and have been developed by the NICUs themselves. | The HDMB can be accessed in the NICU. | Access is initiated when an infant is in need or a mother is medically eligible and willing to donate. The infant can receive HDM until discharge. |

Table 2: Specification of the implementation strategies of Neo-MILK as suggested by Proctor et al. [40]

|  | Training and certification for NICU staff | Provision of manuals on lactation and breastfeeding | Provision of information material for NICU staff | Provision of checklist for prepartal consultation | Increasing commitment through nudging | Provision of SOPs for setting up the HDMB | Financial resource |
| --- | --- | --- | --- | --- | --- | --- | --- |
| Actor(s) | Project coordinator | Project coordinator | Project coordinator | Project coordinator | Project coordinator | Project coordinator | Statutory health insurances  Funder: G-BA |
| Actions(s) | Online-training on the lactation and breastfeeding support intervention | Support through guidelines (instructions for action) | Support through posters on lactation and breastfeeding, and fact sheets | Support through guidelines (conversation guide) | Nudging (e.g. obtaining commitment in written form or public presentation through posters) | Support trough procedural policies | Neo-MILK intervention is part of a funded project of the G-BA, the NICUs get financial support (NICUs receive equipment for setting up the human donor milk bank and funds to hire staff) |
| Target(s) of action | NICU staff and other affiliated departments (if desired) | NICU staff | NICU staff | NICU staff | NICU staff | NICU | NICU |
| Temporality | Beginning of intervention phase in respective NICU | Throughout intervention phase in respective NICU with start at the beginning of intervention phase | Throughout intervention phase in respective NICU with start at the beginning of intervention phase | Throughout intervention phase in respective NICU with start at the beginning of intervention phase | Throughout intervention phase in respective NICU with start at the beginning of intervention phase | Throughout intervention phase in respective NICU with start at the beginning of intervention phase | HDMB equipment: intervention phase,  Infrastructural payments: Beginning of the control phase |
| Dose | 2 h training (crash course), 8 h training (long version) | Initial setup of material, need-based restocking | Initial setup of material, need-based restocking | Initial setup of material, need-based restocking | Initial setup of material, need-based restocking | Initial setup of material, need based restocking | One-time delivery of the HDMB equipment, yearly payments for infrastructural support |
| Implementation outcome(s) affected | Adoption, Implementation (Fidelity) | Adoption, Implementation (Fidelity), Sustainability Reaction/Response (organisation) | Adoption, Implementation (Fidelity) | Implementation (Fidelity) | Adoption, Sustainability | Implementation (Penetration), Sustainability | Adoption, Implementation (Costs) |
| Justification | Educational training is a necessary but insufficient strategy for behaviour change. Building knowledge is important for laying the foundation for the actual application of the intervention components. | Educational materials are important to support the NICU staff learning how to deliver the intervention, which may influence the uptake, delivery and maintenance of the intervention. Distributing educational materials promotes to increase self-efficacy and a positive attitude towards the intervention and supports establishing a pro-implementation attitude. | Information materials are useful for summarizing knowledge for NICU staff about the intended mode of delivering the intervention. | Check lists are important to support the NICU staff in delivering the intervention. It provides guidance and a rough list of essential steps to achieve the function of a specific intervention component. | Nudging could improve the NICU staff’s commitment to be involved in delivering the intervention and engaging in its sustained use. | SOPs and other procedural policies support the NICU staff setting up and embed the intervention into routine practice. | Implementing a new model of care is a highly complex process of change in the routine care of the clinics, which are subject to financial pressure anyway. Financial support is necessary to support the adoption of new interventions like Neo-MILK, to optimize the implementation and prepare its maintenance. |
