# Supplementary material for: Effectiveness of structured lactation support and human donor milk banking in German NICUs: a stepped-wedge cluster-randomized trial
Source: BMC Med. 2026 Sep 11;24:504. doi: 10.1186/s12916-026-05211-1 (PMC13587408; doi:10.1186/s12916-026-05211-1)
Supplement: Supplementary file 2 [file 12916_2026_5211_MOESM2_ESM.docx]

**Effectiveness of Structured Lactation Support and Human Donor Milk Banking in German NICUs: A Stepped-Wedge Cluster Randomized Trial**

**Authors:** Juliane Köberlein-Neu¹, Nicola Gabriela Dymek¹, Viola Zimmer¹, Trutz Bommhardt^1^, Anna Katharina Stirner², Isabella Güldenring^4^, Tim Ohnhäuser³, Daniel Wiesen²^,5^, Till Dresbach^6^, Nadine Scholten³^,4^; the Neo-MILK Consortium

**Affiliations:** ¹Centre for Health Economics and Health Services Research, University of Wuppertal, Wuppertal, Germany; ²Department of Business Administration and Health Care Management, University of Cologne, Cologne, Germany; ³Department of Health Services Research, University of Cologne, Cologne, Germany; ⁴Department for Psychosomatic Medicine and Psychotherapy, Centre for Health Services Research and Communication (CHSR), University Hospital Bonn, Bonn, Germany; ^5^Erasmus School of Health Policy & Management (ESHPM), Erasmus University Rotterdam, Rotterdam, Netherlands; ^6^Department of Neonatology, University Hospital Bonn, Bonn, Germany

**Additional File 3**

**Health Economic Evaluation**

The health economic evaluation encompassed three distinct topics: (1) direct intervention costs from the healthcare provider perspective, capturing all resources required for service delivery; (2) implementation costs associated with the initial introduction and integration of the intervention into routine clinical practice; and (3) out-of-pocket costs incurred by parents of very low birth weight infants (birth weight <1,500g), representing the societal burden of care. Additionally, a budget impact analysis (BIA) was conducted to project the financial implications of nationwide implementation from the German statutory health insurance perspective, providing critical information for healthcare policy decision-making.

1. **Assessment of Direct Intervention Costs**

**Methodological Approach**

The calculation of direct intervention costs employed Time-Driven Activity-Based Costing (TDABC) methodology as described by Kaplan and Anderson (2003) [50]. This approach enables precise and realistic cost capture by documenting individual process activities in detail and linking them to specific resource consumption. The TDABC method was selected for its ability to accurately reflect the complexity of healthcare service delivery and its demonstrated validity in hospital settings.

**Data Collection Process**

A systematic multi-stage data collection approach was implemented:

**Stage 1: Qualitative Process Mapping** Semi-structured interviews were conducted with key project team members from five strategically selected NICUs among the 15 participating clinics. Selection criteria ensured representation of diverse hospital characteristics including size, geographic location (urban vs. rural), and organisational structure. Each interview participant held strategic and/or operational responsibilities for intervention implementation and delivery.

Interviews were conducted via Zoom video conferencing platform with audio recording for subsequent analysis. The interview guide was developed deductively based on existing project documentation and intervention delivery protocols. All interviews were transcribed using F4 transcript software, manually verified for accuracy and completeness, and imported into MAXQDA (Version 24.3) for qualitative analysis. Process steps were initially coded deductively based on the interview guide structure, then supplemented with inductively derived codes emerging from the data. All transcripts were coded by a single researcher with initial coding frameworks discussed and validated within the research team. The resulting process map comprehensively documented all intervention activities and associated resource requirements.

**Stage 2: Quantitative Resource Consumption Survey** Based on the process map, a standardised paper-based questionnaire was developed to quantify resource consumption across all participating NICUs. To ensure anonymity while maintaining data linkability, questionnaires were identified only by clinic IDs, enabling pseudonymised responses at the organisational level. Pre-paid return envelopes were provided to eliminate financial barriers to participation. The voluntary nature of participation was explicitly stated on the questionnaire cover sheet.

The questionnaire was distributed once following study completion to designated key personnel in each NICU, identified internally by participating hospitals as having comprehensive knowledge of the implementation process. The instrument captured:

- Time requirements for each intervention activity (measured in minutes, hours, weeks, or months depending on activity type)
- Frequency of activity performance
- Types of intervention activities (e.g., prenatal consultations, lactation counselling sessions, HDMB operations)
- Personnel resources by professional category and time investment per activity
- Capital investments in equipment and consumable materials

**Capacity Cost Rate Calculations**

Capacity Cost Rates (CCR) representing the cost per time unit for each resource type were calculated following TDABC principles. This involved:

**Personnel Costs:** Annual gross salaries were determined based on collective bargaining agreements applicable to German public healthcare institutions for 2023. Middle pay grades with average experience levels were selected to reflect typical staffing profiles across participating NICUs. For physician salaries, averages were calculated between university and municipal hospital rates to account for institutional variation. Gross salaries were adjusted for employer social security contributions (29% of gross salary in 2023, per German Federal Statistical Office).

The effective annual working time was set at 95,280 minutes per full-time equivalent, based on national statistics accounting for holidays, sick leave, and calendar effects. For team-based activities, composite CCRs were calculated based on average team composition across participating centres.

**Cost Aggregation:** Individual clinic costs were calculated by multiplying activity-specific CCRs by reported activity durations. Costs for all intervention components were summed to determine total intervention costs per mother. These were then multiplied by the average number of mothers in each NICU during the six-month observation period. Results were averaged across all clinics to determine mean intervention costs per clinic for the standardised six-month period.

2. **Implementation Cost Assessment**

**Methodological Approach**

Implementation costs, defined as one-time investments required to introduce and integrate the intervention into routine clinical practice, were assessed using the same multi-stage approach as intervention costs. The Stages of Implementation Completion (SIC) framework [51] guided systematic documentation of implementation activities across eight stages:

1. Engagement preparation and feasibility assessment
2. Organisational readiness and capacity building
3. Staff recruitment and role definition
4. Training and competency development
5. Pilot testing and process refinement
6. Service delivery initiation
7. Quality monitoring and fidelity assessment
8. Sustainability planning and maintenance

Each stage was mapped to one of three implementation phases: pre-implementation, implementation, and sustainment [52,53].

**Data Collection and Analysis**

Implementation activities were captured through the same interview and survey processes described for intervention costs. The standardised questionnaire was expanded to include:

- Implementation strategy activities (e.g., training sessions, HDMB establishment)
- Duration and frequency of implementation activities
- Personnel time by professional category
- Capital investments in infrastructure and equipment
- Ongoing quality assurance and monitoring requirements

Costs were calculated using the same CCRs developed for intervention costing. Implementation costs were analysed both at individual clinic level and averaged across all participating centres to determine typical implementation investment requirements.

3. **Parent Out-of-Pocket Cost Assessment**

**Data Collection**

Out-of-pocket expenses incurred by parents were collected through self-administered questionnaires distributed at eight weeks postpartum (t_1_) to both intervention and control group participants. An open-text field allowed retrospective reporting of all privately funded expenses related to infant feeding and lactation support. This approach captured the full range of potential expenses without constraining responses to predetermined categories.

**Cost Categorisation and Analysis**

Reported expenses were manually categorised by a researcher into product categories including:

- Breast pump equipment and accessories
- Pump rental fees
- Lactation support products and supplements
- Medications and therapeutic products
- Transportation costs for the delivery of pumped milk
- Storage equipment and supplies
- Professional lactation consulting fees not covered by insurance

Individual item costs were calculated based on reported unit prices and quantities. Data plausibility checks identified and resolved outliers or data entry errors. Descriptive analyses calculated mean out-of-pocket costs per family with standard deviations to characterise cost variability.

4. **Budget Impact Analysis**

**Analytical Framework**

The BIA followed the International Society for Pharmacoeconomics and Outcomes Research (ISPOR) Task Force Principles of Good Practice [54,55]. The analysis projected the net financial impact of replacing current standard care with the Neo-MILK intervention across the German healthcare system.

**Model Structure**

A decision tree model was constructed to represent clinical pathways and associated costs. The model structure included:

**First Decision Node:** Intervention allocation (Neo-MILK intervention vs. standard care)

**First Chance Node:** Nutritional outcome at NICU discharge

- Exclusive MOM feeding
- Non-exclusive MOM feeding (includes partial MOM or exclusive formula feeding)

**Second Chance Node:** Complication occurrence

- No complications
- At least one complication (including necrotizing enterocolitis, bronchopulmonary dysplasia, late-onset sepsis, or retinopathy of prematurity)

Given the study's sample size limitations for detecting differences in specific complication rates, complications were modelled as a binary outcome. This conservative approach likely underestimates potential cost savings from complication reduction.

**Model Parameters**

**Perspective and Time Horizon:**

- Perspective: German statutory health insurance
- Time horizon: Six months, corresponding to the study observation period
- Discounting: Not applied given the short time horizon

**Target Population:** The target population comprised all live-born infants with birth weight <1,500g in Germany for 2023. Population size was derived from the Institute for Quality and Transparency in Healthcare (IQTiG) national perinatal statistics [56]. Stillbirths across relevant weight categories (<500g, 500-749g, 750-999g, 1,000-1,249g, 1,250-1,499g) were subtracted from total births to determine the live-born population. The annual figure was adjusted to six months assuming uniform birth distribution.

A mother-to-infant ratio correction factor was applied to account for multiple births, as mothers receive the intervention while infants experience complications and generate treatment costs. This ratio was calculated from 2023 national birth statistics.

**Clinical Effectiveness Parameters:** Probabilities for nutritional outcomes and complication rates were derived from Neo-MILK study results:

- Probability of exclusive MOM feeding with intervention
- Probability of exclusive MOM feeding with standard care
- Conditional probability of complications given exclusive MOM feeding
- Conditional probability of complications given non-exclusive MOM feeding

Nutritional status at NICU discharge served as a proxy for feeding status during the entire NICU stay, validated through longitudinal feeding data analysis. The BIA was conducted using observed (unadjusted) data without further statistical adjustment

**Cost Parameters:**

*Intervention Costs:* Direct intervention costs from the provider perspective were adjusted by removing employer social security contributions to reflect the payer perspective. Cost-neutral reimbursement by health insurance was assumed.

*Complication Treatment Costs:* Treatment costs were derived from the German DRG catalogue for 2023 [57]. All DRGs applicable to infants <1,500g without significant surgical procedures were identified, as the modelled complications typically require medical rather than surgical management. Average reimbursement including nursing supplements was calculated using standardised DRG calculators.

Mean costs were calculated across all relevant DRGs to determine average complication treatment costs. For the binary complication model, costs for one, two, or three complications were averaged to determine the composite cost for "any complication occurrence."

**Sensitivity Analyses**

Univariate deterministic sensitivity analyses examined the impact of parameter uncertainty on budget impact projections:

**Best-Case Scenario:** Applied the highest exclusive MOM feeding rate achieved in any intervention NICU during the study period (77%), representing optimal implementation conditions.

**Worst-Case Scenario:** Applied the lowest exclusive MOM feeding rate achieved in any intervention NICU (33%), representing challenging implementation conditions.

**Model Validation**

The model structure and parameter selection were validated through:

- Expert clinical review by neonatologists
- Comparison with published economic evaluations of similar interventions
- Sensitivity analyses to test structural assumptions
- Cross-validation of complication costs with actual hospital billing data where available

**Statistical Analysis**

All cost data were analysed descriptively. Individual clinic resource consumption and costs were first determined for each intervention component, then averaged across all clinics to calculate mean costs per clinic within the six-month observation period. Results were reported as means with standard deviations where applicable.

**Ethical and Regulatory Considerations**

The economic evaluation was conducted as an integral component of the Neo-MILK study with approval from the lead ethics committee. Voluntary participation in cost data collection was emphasized, and all data were handled according to data protection regulations. No individual-level cost data were reported to maintain participant confidentiality.

**Results**

*Process Mapping*

Five semi-structured interviews were conducted with key personnel from purposefully selected NICUs, lasting 30-60 minutes each. Participants represented urban (n=2) and rural (n=3) facilities, with four from university hospitals/teaching hospitals and one from a specialised centre. The interview sample included three senior physicians and two nursing leaders.

The resulting process map identified four overarching domains: (1) resources, (2) initial implementation, (3) intervention delivery, and (4) continuous improvement and sustainability. Resource requirements encompassed qualified medical personnel (e.g., lactation consultants, neonatologists), administrative staff, infrastructure for HDMB operations (including HACCP-compliant facilities and cooling systems), and lactation support materials. The initial implementation phase involved stakeholder engagement, feasibility assessment, project team recruitment, staff training, and HDMB establishment. Intervention delivery comprised pre- and postnatal counselling, needs-based follow-up support, HDM counselling, and HDMB operations including collection, testing, processing, documentation, storage, and distribution. Continuous improvement strategies focused on systematic integration of experiences into clinical processes, ongoing training, and network development.

*Intervention and Implementation Costs*

Survey Response and Sample Characteristics

Eight of 15 participating NICUs (53.3%) completed the standardised cost survey. Responding clinics were predominantly urban (87.5%), publicly or non-profit owned (100%), with bed capacity ranging from 451 to 1,675 (mean: 1,002.8). Non-responding clinics showed similar characteristics, suggesting minimal selection bias.

Direct Intervention Costs

The mean total time investment for lactation support intervention delivery was 4.33 hours per mother (SD: 3.53), translating to 101.66 hours (SD: 80.06) per clinic over six months with an average of 23.15 mothers (SD: 11.47). This corresponded to mean total costs of €17,338.37 (SD: €25,025.56) per clinic for the six-month period.

Human Donor Milk Bank Operating Costs

Two of the eight clinics that completed the cost survey established operational HDMBs during the study period, with mean operating duration of 9.5 months (SD: 4.95). Operating costs totalled €2,605.52 (SD: €2,835.74) per clinic over the operational period, comprising:

- Internal departmental coordination: 27.46 hours (€2,576.71; 95.38% of total time)
- Regulatory authority communication: 0.25 hours (€28.82; 4.62% of total time)
- Microbiological testing: Weekly testing via internal (n=1) or external laboratory (€90/test; n=1)

Implementation Time Investment

Implementation activities required substantial time investments across multiple domains, though monetary valuation was not feasible due to imprecise personnel cost specifications. The total implementation effort averaged approximately 1,649 hours per NICU across the study period, representing a major organisational commitment.

Project team coordination consumed the largest share of implementation time, with initial implementation meetings requiring 495.65 hours (SD: 589.36) and ongoing coordination post-establishment demanding an additional 504.83 hours (SD: 630.53) over six months. Nearly half (46%) of meeting time addressed unplanned events, highlighting the adaptive management required during implementation. Project teams comprised an average of 7.00 members (SD: 3.18), typically including 2.5 physicians, 1.5 lactation consultants, 0.67 nursing leaders, and 1.0 hygiene specialist, reflecting the multidisciplinary nature of the intervention.

HDMB establishment represented the second major time investment, requiring 377.58 hours (SD: 461.95) over an average of 11.25 months (SD: 5.95). The majority of this time (71%) was devoted to developing HACCP concepts and standard operating procedures (267.54 hours; SD: 366.75), essential for ensuring food safety compliance. Internal departmental coordination consumed an additional 76.41 hours (SD: 99.64), while regulatory interactions and inspections required 33.63 hours (SD: 43.70). Notably, only two of eight surveyed clinics successfully established operational HDMBs during the study period, with establishment times ranging from 6 to 12 months.

Staff training and sensitisation activities collectively required 270.93 hours per clinic. Formal training programs accounted for 102.63 hours (SD: 97.58), with short-format sessions (2 hours) reaching an average of 37 staff members and consuming 74.13 hours of total staff time, while intensive long-format training (9 hours) engaged fewer participants (mean: 3.56) but required 28.5 hours. Staff sensitisation efforts, aimed at creating awareness and behavioural activation across the broader hospital workforce, demanded 168.30 hours (SD: 273.63) per clinic. The nursing staff orientation was particularly time-intensive at 121.17 hours (SD: 245.03), reflecting the central role of nurses in intervention delivery. Medical staff orientation (13.40 hours) and general behavioural activation strategies (20.06 hours) required comparatively less time but remained essential for creating a supportive implementation climate.

The substantial variation in time requirements across clinics (evidenced by large standard deviations) suggests that implementation efficiency was influenced by local contextual factors, organisational readiness, and prior experience with similar interventions. The time investment data underscore that successful implementation of complex lactation support interventions requires significant organisational commitment beyond direct service delivery costs.

*Out-of-Pocket Costs*

Parent-reported out-of-pocket expenses were collected from 171 mothers (intervention: n=72; control: n=99) at 8 weeks postpartum, revealing comparable financial burdens between groups despite the intervention's lactation support components.

The mean total out-of-pocket costs per mother, including those reporting no expenses, were €145.52 (SD: 176.73) in the intervention group and €156.07 (SD: 209.35) in the control group, representing a non-significant difference of -€10.55 (95% CI: -69.00 to 47.90). When analysis was restricted to mothers reporting any expenses (76% intervention, 75% control), mean costs increased to €190.50 (SD: 179.33) and €208.80 (SD: 218.35) respectively, with a similar between-group difference of -€18.30 (95% CI: -78.45 to 42.85).

Breast pump-related expenses dominated out-of-pocket costs in both groups, accounting for approximately 60% of total expenses among intervention mothers and 49% among controls. These costs comprised three components: pump purchase or rental (intervention: €58.17; control: €43.40), accessories such as flanges and collection bottles (intervention: €10.35; control: €20.94), and rental fees or deposits (intervention: €18.83; control: €12.17). The higher pump-related expenses in the intervention group (€86.54 vs. €76.50) may reflect increased pumping frequency due to enhanced lactation support, though 40% of intervention and 46% of control mothers reported no pump-related expenses, suggesting variable insurance coverage or hospital provision.

Nutritional supplements showed an interesting inverse pattern, with control mothers spending more than twice as much (€15.87 vs. €6.29) on products such as lactation teas, fenugreek capsules, and other galactagogues. This difference was primarily driven by higher use of alternative lactation support products in the control group (12% reporting expenses vs. 21%), potentially reflecting attempts to compensate for less structured professional support. Similarly, control mothers reported higher expenses for medications and therapeutic interventions (€11.44 vs. €8.88), including antibiotics for mastitis and complementary therapies like acupuncture.

Other expense categories showed more uniform distributions between groups. Breastfeeding clothing expenses were reported by approximately 15% of mothers in both groups, though intervention mothers spent more when purchases were made (€77.31 vs. €66.69 among purchasers). Essential supplies such as breast pads (intervention: €4.96; control: €4.80), nipple care products (intervention: €2.93; control: €4.05), and milk storage bags (intervention: €2.21; control: €3.36) represented minor but consistent expenses across both groups. Sterilisation equipment, bottles, and feeding accessories collectively added €12-20 per mother.

The substantial variation in individual expenses (ranging from €0 to €709 in the intervention group and €0 to €1,141 in controls) reflected differences in insurance coverage, personal resources, and lactation challenges. Notably, the highest individual expenses in both groups were associated with prolonged pump rental periods and purchase of hospital-grade pumps, highlighting the financial vulnerability of mothers experiencing significant lactation difficulties.

The comparable out-of-pocket costs between groups suggest that the intervention did not substantially reduce parents' financial burden despite potentially improving lactation outcomes. This finding has important implications for intervention design, suggesting that comprehensive lactation support programs should consider mechanisms to address equipment costs, which represent the primary financial barrier for families.

*Budget Impact Analysis*

The BIA projected the financial implications of implementing the Neo-MILK intervention across the German healthcare system, indicating potential for modest cost savings despite upfront intervention investments.

Target Population and Model Structure

The analysis targeted all live-born infants with birth weight <1,500g in Germany, totalling 7,818 annual births based on 2023 national perinatal statistics. After excluding 1,625 stillbirths across weight categories (<500g: 367; 500-749g: 541; 750-999g: 314; 1,000-1,499g: 403), the six-month target population comprised 3,909 infants, assuming uniform birth distribution throughout the year.

The decision tree model captured the intervention's impact through two sequential nodes: nutritional status at NICU discharge (exclusive vs. non-exclusive MOM feeding) and subsequent complication occurrence. This binary complication approach, while conservative, was necessitated by sample size limitations for detecting differences in specific complication rates. The model incorporated a mother-to-infant ratio correction factor to account for multiple births, as mothers receive the intervention while infants generate treatment costs.

Cost Parameters and Clinical Effectiveness

Intervention costs of €649 per infant were derived from the maternal intervention costs (€17,338 per clinic/23.15 mothers = €749 per mother), adjusted for the mother-to-infant ratio and excluding employer social security contributions to reflect the payer perspective. This assumes cost-neutral reimbursement by statutory health insurance.

Complication treatment costs were calculated from six relevant DRGs for infants <1,500g without surgical procedures, ranging from €47,326 (P64Z: 1,250-1,499g) to €168,649 (P61B: <600g). The weighted mean complication cost of €107,888 (SD: €60,761) was doubled to €215,775 to account for the occurrence of multiple complications, based on study observations that affected infants typically experienced two or more complications.

Clinical effectiveness parameters from the Neo-MILK study showed exclusive MOM feeding rates of 51% with intervention versus 44% with standard care (unadjusted values). Complication rates were 22% among exclusively MOM-fed infants and 24% among non-exclusively MOM-fed infants (unadjusted values). These descriptive rates informed the branching probabilities of the decision-tree model and should not be interpreted as an estimate of a causal effect.

**Base Case Results**

The base case analysis revealed total six-month healthcare costs of €194,199,006 for the intervention pathway versus €195,009,182 for standard care, yielding net savings of €810,176 (€207 per infant). This 0.4% reduction in total costs occurred despite the additional intervention investment and is attributable in the model to the 7-percentage-point improvement in unadjusted exclusive MOM feeding rates^[1]^ and the consequent reduction in costly complications.

The expected cost per infant was €49,680 with intervention versus €49,887 with standard care. Breaking down the intervention pathway: 11% of infants achieved exclusive MOM feeding without complications (cost: €649), 40% achieved exclusive feeding but experienced complications (cost: €216,424), 12% had non-exclusive MOM feeding without complications (cost: €649), and 37% had non-exclusive MOM feeding with complications (cost: €216,424). The reduction in the highest-cost category (non-exclusive MOM feeding with complications: 37% intervention vs. 43% control) primarily drove the cost savings.

**Sensitivity Analysis**

The economic viability of the intervention was highly sensitive to achieved exclusive MOM feeding rates. In the best-case scenario, achieving the highest observed study rate of 77% exclusive MOM feeding generated substantial savings of €2,828,246 over six months. This scenario reduced the proportion of infants in high-cost complication pathways from 49% to 23%, with 60% achieving exclusive MOM feeding without complications.

Conversely, the worst-case scenario using the lowest observed rate of 33% exclusive MOM feeding resulted in net costs of €1,888,021. In this scenario, 67% of infants remained in high-cost pathways, negating any savings from complication reduction. The threshold for cost-neutrality occurred at approximately 44% exclusive MOM feeding with intervention, equivalent to the control group's baseline rate.

**Implications for Healthcare Policy**

The budget impact findings suggest that nationwide implementation of structured lactation support could generate modest healthcare savings while improving infant health outcomes. The €810,176 savings over six months, while representing only 0.4% of total NICU costs for this population, would accumulate to €1.62 million annually. These savings exclude potential longer-term benefits from reduced readmissions, improved neurodevelopmental outcomes, and decreased chronic disease risk associated with human milk feeding.

The sensitivity analysis underscores that economic benefits depend critically on implementation quality. Centres achieving high exclusive feeding rates (>60%) could generate savings exceeding €700 per infant, while poor implementation could increase costs by up to €480 per infant. This variation emphasizes the importance of adequate training, resources, and organisational support to ensure effective implementation.

From a health insurance perspective, the intervention appears economically viable given the conservative modelling assumptions. The analysis likely underestimates savings by modelling complications binarily, excluding long-term benefits, and assuming full intervention costs despite potential economies of scale. Moreover, the non-monetary benefits of increased maternal satisfaction, reduced family stress, and improved maternal-infant bonding provide additional value beyond the economic analysis.

1. The adjusted trial estimate reported in the main manuscript is 11 percentage points.
