# Supplementary material for: Effectiveness of structured lactation support and human donor milk banking in German NICUs: a stepped-wedge cluster-randomized trial
Source: BMC Med. 2026 Sep 11;24:504. doi: 10.1186/s12916-026-05211-1 (PMC13587408; doi:10.1186/s12916-026-05211-1)
Supplement: Supplementary file 3 [file 12916_2026_5211_MOESM3_ESM.docx]

**Effectiveness of Structured Lactation Support and Human Donor Milk Banking in German NICUs: A Stepped-Wedge Cluster Randomized Trial**

**Authors:** Juliane Köberlein-Neu¹, Nicola Gabriela Dymek¹, Viola Zimmer¹, Trutz Bommhardt^1^, Anna Katharina Stirner², Isabella Güldenring^4^, Tim Ohnhäuser³, Daniel Wiesen²^,5^, Till Dresbach^6^, Nadine Scholten³^,4^; the Neo-MILK Consortium

**Affiliations:** ¹Centre for Health Economics and Health Services Research, University of Wuppertal, Wuppertal, Germany; ²Department of Business Administration and Health Care Management, University of Cologne, Cologne, Germany; ³Department of Health Services Research, University of Cologne, Cologne, Germany; ⁴Department for Psychosomatic Medicine and Psychotherapy, Centre for Health Services Research and Communication (CHSR), University Hospital Bonn, Bonn, Germany; ^5^Erasmus School of Health Policy & Management (ESHPM), Erasmus University Rotterdam, Rotterdam, Netherlands; ^6^Department of Neonatology, University Hospital Bonn, Bonn, Germany

**Additional File 4**

**Implementation metrics**

**Detailed implementation metrices**

**Coverage**

The intervention achieved full coverage across all 15 participating NICUs and reached an estimated 671 mothers in the intervention group (versus 655 controls). Among the 831 intervention infants, 65% were singletons with a mean gestational age of 28.85 weeks (SD 2.94) and a CRIB score of 3.84 (SD 3.93). Table 1 and 2 display population characteristics by observation period and randomized sequence.

Mothers' demographic characteristics were comparable across groups, with the majority aged 26-40 years (Table 3).

Originally, both Level I and Level II NICUs were intended to be included. However, only Level I centres could be recruited during the enrolment phase. To assess the structural comparability of participating facilities, publicly available hospital quality reports from 2022 (according to §136b SGB V) were analysed. Structural indicators extracted from these reports, such as ownership, total hospital bed capacity, number of neonatal inpatients, and full-time equivalent (FTE) medical and nursing staff, were compared with nationwide averages from all 156 Level I NICUs in Germany. The 15 participating centres showed a heterogeneous composition regarding these parameters but were overall structurally comparable to the national reference group. Slightly higher mean hospital bed counts and neonatal inpatient volumes were observed but did not suggest systematic bias. Consequently, the study sample can be considered broadly representative of German Level I NICUs, though generalization to other Level units is limited due to their absence in the study sample.

Table 1: Characteristics of infants displayed by observation period

|  | Period 1 | Period 2 | Period 3 | Period 4 | Total |
| --- | --- | --- | --- | --- | --- |
| Children | 402 (100.00%) | 416 (100.00%) | 428 (100.00%) | 381 (100.00%) | 1,627 (100.00%) |
| Female | 185 (46.25%) | 195 (47.10%) | 220 (51.64%) | 188 (49.47%) | 788 (48.64%) |
| Multiple birth | 153 (38.06%) | 130 (31.25%) | 137 (32.01%) | 136 (35.70%) | 556 (34.17%) |
| Malformations | 48 (12.15%) | 41 (9.98%) | 58 (13.65%) | 36 (9.50%) | 183 (11.37%) |
| CRIB-Score [mean (SD)] | 3.91 (3.81) | 4.32 (4.22) | 3.63 (3.98) | 3.76 (3.77) | 3.91 (3.96) |
| **Mode of delivery**  Spontaneous birth | 37 (9.20%) | 50 (12.02%) | 32 (7.48%) | 26 (6.82%) | 1  45 (8.91%) |
| Caesarean | 326 (81.09%) | 328 (78.85%) | 348 (81.31%) | 317 (83.20%) | 1,319 (81.07%) |
| Emergency caesarean | 37 (9.20%) | 32 (7.69%) | 43 (10.05%) | 36 (9.45%) | 148 (9.10%) |
| **Birth weight**  < 500g | 21 (5.22%) | 28 (6.73%) | 12 (2.80%) | 24 (6.30%) | 85 (5.22%) |
| 500–749g | 81 (20.15%) | 74 (17.79%) | 80 (18.69%) | 63 (16.54%) | 298 (18.32%) |
| 750–999g | 96 (23.88%) | 93 (22.36%) | 94 (21.96%) | 87 (22.83%) | 370 (22.74%) |
| 1000–1249g | 99 (24.63%) | 99 (23.80%) | 97 (22.66%) | 92 (24.15%) | 387 (23.79%) |
| 1250–1499g | 105 (26.12%) | 118 (28.37%) | 145 (33.88%) | 113 (29.66%) | 481 (29.56%) |
| **Reason for Preterm birth**  Contractions | 145 (36.07%) | 144 (34.62%) | 128 (29.91%) | 105 (27.56%) | 522 (32.08%) |
| Clinical chorioamnionitis | 33 (8.23%) | 52 (12.50%) | 54 (12.65%) | 40 (10.50%) | 179 (11.02%) |
| Preeclampsia | 50 (12.44%) | 45 (10.82%) | 63 (14.72%) | 61 (16.01%) | 219 (13.46%) |
| HELLP | 15 (3.73%) | 17 (4.09%) | 22 (5.15%) | 18 (4.72%) | 72 (4.43%) |
| Pathologic CTG | 69 (17.16%) | 83 (19.95%) | 107 (25.00%) | 92 (24.15%) | 351 (21.57%) |
| IUGR | 90 (22.39%) | 65 (15.62%) | 104 (24.30%) | 82 (21.52%) | 341 (20.96%) |
| Anhydramnios | 4 (1.00%) | 5 (1.20%) | 7 (1.64%) | 4 (1.05%) | 20 (1.23%) |
| Rupture of the membranes | 35 (8.71%) | 30 (7.21%) | 27 (6.31%) | 26 (6.82%) | 118 (7.25%) |
| Placenta abruption | 15 (3.73%) | 25 (6.01%) | 28 (6.54%) | 24 (6.30%) | 92 (5.65%) |

Note: CRIB = Clinical Risk Index for Babies; CTG = Cardiotocography; HELLP = Hemolysis, Elevated Liver Enzymes, and Low Platelet Count; IUGR = Intrauterine Growth Restriction; NICU = Neonatal Intensive Care Unit; SD = Standard Deviation; Clinical chorioamnionitis was recorded clinically at birth (including suspected cases) as an indication for preterm birth.

Tabel 2: Characteristics of infants in Period 1 displayed by randomization sequence

| Variable | Sequence 1 | Sequence 2 | Sequence 3 | Total |
| --- | --- | --- | --- | --- |
| Children | 150 (100.00%) | 110 (100.00%) | 142 (100.00%) | 402 (100.00%) |
| Female | 75 (50.00%) | 42 (38.18%) | 68 (48.57%) | 185 (46.25%) |
| Multiple Birth | 63 (42.00%) | 33 (30.00%) | 57 (40.14%) | 153 (38.06%) |
| Malformations | 14 (9.40%) | 22 (20.56%) | 12 (8.63%) | 48 (12.15%) |
| CRIB Score [mean (SD)] | 4.59 (4.09) | 2.74 (2.98) | 4.12 (3.89) | 3.91 (3.81) |
| **Mode of Delivery**  Emergency Caesarean | 4 (2.67%) | 10 (9.09%) | 23 (16.20%) | 37 (9.20%) |
| Caesarean | 125 (83.33%) | 85 (77.27%) | 116 (81.69%) | 326 (81.09%) |
| Spontaneous Delivery | 21 (14.00%) | 15 (13.64%) | 1 (0.70%) | 37 (9.20%) |
| **Birth Weight**  <500g | 8 (5.33%) | 4 (3.64%) | 9 (6.34%) | 21 (5.22%) |
| 500-749g | 37 (24.67%) | 15 (13.64%) | 29 (20.42%) | 81 (20.15%) |
| 750-999g | 39 (26.00%) | 19 (17.27%) | 38 (26.76%) | 96 (23.88%) |
| 1000-1249g | 27 (18.00%) | 35 (31.82%) | 37 (26.06%) | 99 (24.63%) |
| 1250-1499g | 39 (26.00%) | 37 (33.64%) | 29 (20.42%) | 105 (26.12%) |
| **Reason for Preterm Birth**  Clinical chorioamnionitis | 9 (6.00%) | 16 (14.55%) | 8 (5.67%) | 33 (8.23%) |
| Anhydramnios | 0 (0.00%) | 3 (2.73%) | 1 (0.70%) | 4 (1.00%) |
| Rupture of the membranes | 12 (8.00%) | 11 (10.00%) | 12 (8.45%) | 35 (8.71%) |
| HELLP | 2 (1.33%) | 9 (8.18%) | 4 (2.82%) | 15 (3.73%) |
| IUGR | 36 (24.00%) | 33 (30.00%) | 21 (14.79%) | 90 (22.39%) |
| Placental Abruption | 5 (3.33%) | 5 (4.55%) | 5 (3.52%) | 15 (3.73%) |
| Preeclampsia | 18 (12.00%) | 18 (16.36%) | 14 (9.86%) | 50 (12.44%) |
| Contractions | 61 (40.67%) | 50 (45.45%) | 34 (23.94%) | 145 (36.07%) |
| Pathological CTG | 26 (17.33%) | 23 (20.91%) | 20 (14.08%) | 69 (17.16%) |

Note: CRIB = Clinical Risk Index for Babies; CTG = Cardiotocography; HELLP = Hemolysis, Elevated Liver Enzymes, and Low Platelet Count; IUGR = Intrauterine Growth Restriction; NICU = Neonatal Intensive Care Unit; SD = Standard Deviation; Clinical chorioamnionitis was recorded clinically at birth (including suspected cases) as an indication for preterm birth.

Table 3: Characteristics of mothers, responding to the survey

|  | Total | Control | Intervention | SMD |
| --- | --- | --- | --- | --- |
| Number | 290 (100.00%) | 163 (100%) | 127 (100%) |  |
| Native language German | 249 (85.86%) | 134 (82,21%) | 115 (90,55%) | 0.25 |
| Relationship with shared household | 267 (92.07%) | 148 (90,80%) | 119 (93,70%) | 0.11 |
| **Form of education** No graduation | 2 (0.69%) | 2 (1,23%) | 0 (0,00%) | 0.16 |
| 9 years of education | 14 (4.83%) | 9 (5,52%) | 5 (3,94%) | 0.07 |
| 10 years of education | 50 (17.24%) | 29 (17,79%) | 21 (16,54%) | 0.03 |
| 12 years of education | 60 (20.69%) | 32 (19,63%) | 28 (22,05%) | 0.06 |
| University/college | 158 (54.48%) | 86 (52,76%) | 72 (56,69%) | 0.08 |

Note: SMD = standardised mean difference between groups; SMD <0.10 negligible, <0.20 small.

**Characteristics of NICU staff participating in the survey**

Table 4: Characteristics of NICU staff at the two data collection time points *"Beginning of intervention phase"* and *"After end of intervention phase"*

|  | **Beginning of intervention phase** | **After end of intervention phase** | SMD |
| --- | --- | --- | --- |
| Number of responses | 259 | 66 |  |
| **Sex** | 236 (92.3%) | 58 (84.1%) |  |
| Male | 24 (10.2%) | 3 (5.2%) | 0.19 |
| Female | 211 (89.4%) | 55 (94.8%) | 0.20 |
| Diverse | 1 (0.4%) | - | 0.09 |
| **Age (years)** | 233 (98.6%) | 59 (85.5%) |  |
| < 20 | - | - |  |
| 20–30 | 69 (29.6%) | 13 (22.0%) | 0.17 |
| 31–40 | 66 (28.3%) | 13 (22.0%) | 0.15 |
| 41–50 | 47 (20.2%) | 13 (22.0%) | 0.05 |
| 51–60 | 45 (19.3%) | 16 (27.1%) | 0.19 |
| 61–65 | 5 (2.2%) | 4 (6.9%) | 0.23 |
| > 65 | 1 (0.4%) | - | 0.09 |
| **Participation in continuing education** | 230 (88.8%) | 53 (76.8%) |  |
| Every 1–2 weeks | 17 (7.4%) | 7 (13.2%) | 0.19 |
| Approx. once per month | 46 (20.0%) | 10 (18.9%) | 0.03 |
| Approx. once per quarter | 89 (38.7%) | 17 (32.1%) | 0.14 |
| Approx. once every six months | 43 (18.7%) | 14 (26.4%) | 0.19 |
| Approx. once per year | 22 (9.6%) | 3 (5.6%) | 0.15 |
| Never | 3 (1.3%) | 1 (1.9%) | 0.05 |
| Other | 10 (4.3%) | 1 (1.9%) | 0.14 |
| **Professional role** | 232 (89.2%) | 59 (85.5%) |  |
| Physician | 72 (31.0%) | 14 (23.7%) | 0.16 |
| Nurse | 152 (65.6%) | 42 (71.2%) | 0.12 |
| Other | 8 (3.4%) | 3 (5.1%) | 0.08 |
| **Employment type** | 236 (90.8%) | 59 (85.5%) |  |
| Full-time | 131 (55.5%) | 33 (55.9%) | 0.01 |
| Part-time (> 50%) | 67 (28.4%) | 20 (33.9%) | 0.12 |
| Part-time (= 50%) | 20 (8.5%) | 3 (5.1%) | 0.14 |
| Part-time (< 50%) | 18 (7.6%) | 3 (5.1%) | 0.10 |
| **Professional experience (years)** | 235 (90.4%) | 59 (85.5%) |  |
| < 5 | 42 (17.9%) | 7 (11.9%) | 0.17 |
| 5–10 | 57 (24.3%) | 8 (13.6%) | 0.28 |
| 11–15 | 36 (15.3%) | 12 (20.2%) | 0.13 |
| 16–20 | 18 (7.7%) | 6 (10.2%) | 0.09 |
| > 20 | 82 (34.8%) | 26 (44.1%) | 0.19 |

Note: SMD = standardised mean difference between groups; SMD <0.10 negligible, <0.20 small.

**Acceptability, Appropriateness, and Feasibility**

Pre-implementation assessment among 174 NICU staff (pre-implementation assessment includes only responses of those familiar with intervention content) showed high acceptability (83-92% agreement across items), with staff endorsing, welcoming, and approving the intervention components. The intervention was deemed appropriate for mothers of preterm infants, with 83-87% rating components as suitable, fitting, and applicable. Feasibility scores were somewhat lower but still positive, with 70-85% rating components as possible, doable, implementable, and easy to use.

**Intervention Fidelity**

Intervention materials showed differential uptake: information flyers achieved 100% adoption across NICUs, posters 93.3%, mother bags 80%, and prepartal counselling guides 66.7%. Core intervention components showed high adherence with context-specific adaptations. Prepartal counselling followed protocol in 71% of cases, reaching an average of 8.4/10 eligible mothers per site. Protocol deviations primarily involved distributing content across multiple sessions or delivery by different professionals due to time constraints or parental emotional states. Information materials reached 8.4/10 parents through intended channels (72% adherence), with posters displayed as planned in 74% of units. Service quality remained high, with adaptations serving to improve parent engagement rather than compromising core components. Maternal responsiveness was strongly positive, with >95% understanding provided information and 96% feeling taken seriously by NICU staff.

**Implementation Fidelity**

Implementation support materials showed variable adoption: pocket guides (100%), one-minute-wonder posters (93.3%), prepartal counselling checklists (80%), and commitment posters (33.3%). Despite universal availability, only 51% of staff reported receiving training invitations, with actual participation at 60% for short sessions and 11% for comprehensive training. The intervention handbook reached 61% of staff, with 67% actively using it. Practical tool utilisation varied: checklists (61% of recipients), pocket guides (30%), and identification buttons (29%). Service quality for implementation strategies remained high, with dose delivered varying by strategy but maintaining adherence to core principles. Staff responsiveness to implementation supports was moderate, suggesting room for improved dissemination strategies.

**Sustainability**

Sustainability indicators assessed through multiple validated scales showed promising results. The NoMAD scale (Normalisation Measure Development Questionnaire [46]) revealed favourable normalisation conditions from intervention onset (coherence >77%, cognitive participation >95%, collective action >80%, reflexive monitoring positive). Post-intervention assessment indicated integration: 75% developed shared understanding, 87% recognised practical benefits, and 86% identified implementation champions.

The PRESS (Provider REport of Sustainment Scale [47]) scale indicated 69% of staff applied intervention components whenever possible, 66% maintained application despite changing conditions, and 61% integrated components into routine care. The SMSS (Sustainment Measurement System Scale [48]) revealed strong alignment with organisational values (87%), supportive networks (79%), high staff engagement (67%), and emerging continuation plans (59%). These combined indicators suggest robust potential for long-term sustainability across participating NICUs.
