# Supplementary material for: Effectiveness of structured lactation support and human donor milk banking in German NICUs: a stepped-wedge cluster-randomized trial
Source: BMC Med. 2026 Sep 11;24:504. doi: 10.1186/s12916-026-05211-1 (PMC13587408; doi:10.1186/s12916-026-05211-1)
Supplement: Supplementary file 4 [file 12916_2026_5211_MOESM4_ESM.docx]

**Effectiveness of Structured Lactation Support and Human Donor Milk Banking in German NICUs: A Stepped-Wedge Cluster Randomized Trial**

**Authors:** Juliane Köberlein-Neu¹, Nicola Gabriela Dymek¹, Viola Zimmer¹, Trutz Bommhardt^1^, Anna Katharina Stirner², Isabella Güldenring^4^, Tim Ohnhäuser³, Daniel Wiesen²^,5^, Till Dresbach^6^, Nadine Scholten³^,4^; the Neo-MILK Consortium

**Affiliations:** ¹Centre for Health Economics and Health Services Research, University of Wuppertal, Wuppertal, Germany; ²Department of Business Administration and Health Care Management, University of Cologne, Cologne, Germany; ³Department of Health Services Research, University of Cologne, Cologne, Germany; ⁴Department for Psychosomatic Medicine and Psychotherapy, Centre for Health Services Research and Communication (CHSR), University Hospital Bonn, Bonn, Germany; ^5^Erasmus School of Health Policy & Management (ESHPM), Erasmus University Rotterdam, Rotterdam, Netherlands; ^6^Department of Neonatology, University Hospital Bonn, Bonn, Germany

**Additional File 5**

**Secondary effectiveness outcomes**

**Infant outcomes**

Table 1: Secondary effectiveness outcomes at the infant level: multilevel regression analyses for feeding modes, complications, HDM use, and length of stay

Note: MOM = Mother’s Own Milk; HDM = Human Donor Milk; CI = Confidence Interval; ICC = Intraclass Correlation Coefficient; NICU = Neonatal Intensive Care Unit; SD = Standard Deviation

Table 2: Secondary effectiveness outcomes at the infant level: multilevel regression analyses length of stay unadjusted and adjusted for gestational age

Note: CI = Confidence Interval; ICC = Intraclass Correlation Coefficient; NICU = Neonatal Intensive Care Unit; SD = Standard Deviation; GA = gestational age

Figure 1: Site-specific intervention effects on the primary endpoint

**Maternal outcomes**

Table 3: Regression results for maternal secondary outcomes, including lactation initiation, willingness to donate human milk, and willingness to accept human milk

Note: MOM = Mother’s Own Milk; HDM = Human Donor Milk; CI = Confidence Interval; ICC = Intraclass Correlation Coefficient; NICU = Neonatal Intensive Care Unit; SD = Standard Deviation

Table 4: Multilevel regression analyses of two maternal secondary outcomes: breast-feeding self-efficacy and the amount of milk pumped 14 days postpartum

Note: CI = Confidence Interval; ICC = Intraclass Correlation Coefficient; NICU = Neonatal Intensive Care Unit; p.p = postpartum; SD = Standard Deviation

Table 5: Multilevel logistic regression analyses of the maternal secondary outcome amount of milk pumped 14 days postpartum (reaching the 500ml threshold 14d pp) and its association with birth weight

Note: CI = Confidence Interval; ICC = Intraclass Correlation Coefficient; NICU = Neonatal Intensive Care Unit; SD = Standard Deviation
