# Supplementary material for: Effectiveness of structured lactation support and human donor milk banking in German NICUs: a stepped-wedge cluster-randomized trial
Source: BMC Med. 2026 Sep 11;24:504. doi: 10.1186/s12916-026-05211-1 (PMC13587408; doi:10.1186/s12916-026-05211-1)
Supplement: Supplementary file 5 [file 12916_2026_5211_MOESM6_ESM.docx]

**Effectiveness of Structured Lactation Support and Human Donor Milk Banking in German NICUs: A Stepped-Wedge Cluster Randomized Trial**

**Authors:** Juliane Köberlein-Neu¹, Nicola Gabriela Dymek¹, Viola Zimmer¹, Trutz Bommhardt^1^, Anna Katharina Stirner², Isabella Güldenring^4^, Tim Ohnhäuser³, Daniel Wiesen²^,5^, Till Dresbach^6^, Nadine Scholten³^,4^; the Neo-MILK Consortium

**Affiliations:** ¹Centre for Health Economics and Health Services Research, University of Wuppertal, Wuppertal, Germany; ²Department of Business Administration and Health Care Management, University of Cologne, Cologne, Germany; ³Department of Health Services Research, University of Cologne, Cologne, Germany; ⁴Department for Psychosomatic Medicine and Psychotherapy, Centre for Health Services Research and Communication (CHSR), University Hospital Bonn, Bonn, Germany; ^5^Erasmus School of Health Policy & Management (ESHPM), Erasmus University Rotterdam, Rotterdam, Netherlands; ^6^Department of Neonatology, University Hospital Bonn, Bonn, Germany

**Additional File 1**

**Reporting Standards: StaRI and CONSORT 2025 + SWD-Extension**

## Table 1: StaRI Checklist

*Standards for Reporting Implementation Studies (Pinnock et al., BMJ 2017;356:i6795)*

| **Item** | **Checklist Item** | **Location (Page / Line)** |
| --- | --- | --- |
| **Title and Abstract** | | |
| **1** | Identification as an implementation study, and description of the methodology in the title and/or keywords | p. 1 (title); p. 3, l. 65 (abstract: hybrid type 1 effectiveness-implementation trial) |
| **2** | Abstract: identification as implementation study with description of implementation strategy, intervention, key implementation and health outcomes | pp. 3–4, l. 62–97 (structured abstract) |
| **Introduction** | | |
| **3** | Description of the problem, challenge or deficiency in healthcare that the intervention aims to address | pp. 5–7, section 1, l. 106–177 |
| **4** | Rationale: scientific background including underpinning theory/framework, pilot work | pp. 5–7, section 1 (refs [20,21] implementation frameworks; [29] formative work); Additional file 2 (Proctor specification) |
| **5** | Aims of the study, differentiating implementation objectives and intervention objectives | p. 7, l. 178–189 (three objectives) |
| **Methods: Description** | | |
| **6** | Design and key features of the evaluation; changes to protocol | pp. 8–9, section 2, l. 192–227; p. 9, l. 231–233 (Level II sites not recruited) |
| **7** | Context in which the intervention was implemented | p. 9, section 2.1, l. 229–233; Additional file 4 (structural representativeness) |
| **8** | Targeted sites: characteristics, eligibility criteria / Target population and eligibility criteria | p. 9, section 2.1, l. 229–241; Additional file 4 (Tables 1–4) |
| **9** | Description of the implementation strategy / Description of the intervention | pp. 9–10, section 2.3, l. 253–270; Additional file 2 (TIDieR and Proctor tables) |
| **10** | Sub-groups recruited for additional research tasks and/or nested studies | p. 9, section 2.1, l. 238–241 (staff and maternal surveys); p. 10, section 2.4 |
| **Methods: Evaluation** | | |
| **11** | Defined pre-specified primary and other outcomes; how assessed; targets | pp. 10–11, section 2.4, l. 272–308; Table 1 (p. 11, l. 310); Additional file 5 (full regression models) |
| **12** | Process evaluation objectives and outcomes related to mechanism of strategy | p. 7, l. 188–189 (process evaluation reported separately); Table 1 (fidelity measures) |
| **13** | Methods for resource use, costs, economic outcomes and analysis | p. 11, section 2.4, l. 300–302; Additional file 3 (full methodology) |
| **14** | Rationale for sample sizes | p. 11, section 2.5, l. 312–318 |
| **15** | Methods of analysis (with reasons) | pp. 11–12, section 2.6, l. 320–342 |
| **16** | A priori sub-group analyses | p. 12, section 2.6, l. 326–335 |
| **Results** | | |
| **17** | Proportion recruited and characteristics of recipient population | pp. 13–14, section 3.1, l. 351–376; Table 2 (p. 14, l. 370); Figure 1 (p. 13, l. 356); Additional file 4 |
| **18** | Primary and other outcomes | pp. 14–17, sections 3.2–3.4, l. 377–472; Table 3; Figures 2–3; Additional files 4–5 |
| **19** | Process data mapped to mechanism of strategy | pp. 16–17, section 3.4, l. 444–472; Additional file 4 |
| **20** | Resource use, costs, economic outcomes and analysis | p. 18, section 3.5, l. 476–483; Additional file 3 |
| **21** | Representativeness and outcomes of subgroups | pp. 14–16, sections 3.2–3.3 (subgroup analyses); Additional file 4 (representativeness) |
| **22** | Fidelity to implementation strategy as planned and adaptation | pp. 16–17, section 3.4; pp. 19–21, section 4.1; Additional file 4 |
| **23** | Contextual changes which may have affected outcomes | p. 8, l. 216–217 (no major external disruptions); p. 15, l. 415 (fourth period: increase in HDM use) |
| **24** | All important harms or unintended effects | p. 10, l. 288–290 (definition and ascertainment); p. 15, l. 410–412 (results); pp. 19–20, l. 518–521 (interpretation) |
| **Discussion** | | |
| **25** | Summary of findings, strengths and limitations, comparisons, conclusions and implications | pp. 19–23, sections 4.1–4.3 and section 5, l. 485–639 |
| **26** | Implications: policy, practice, research (including scalability/sustainability) | pp. 22–23, section 4.3, l. 611–624; p. 23, section 5, l. 625–639 |
| **General** | | |
| **27** | Statements on regulatory approvals, registration, protocol, funding, COI | p. 8, l. 218–220 (registration, ethics); pp. 23–25, Declarations, l. 647–707; protocol refs [29,32] |

## Table 2: CONSORT Extension for Stepped-Wedge Cluster Randomised Trials

*Hemming et al., BMJ 2018;363:k1614*

| **Item** | **Checklist Item** | **Location (Page / Line)** |
| --- | --- | --- |
| **Title and Abstract** | | |
| **1a** | Identification as a SW-CRT in the title | p. 1 (title: Stepped-Wedge Cluster-Randomized Trial) |
| **1b** | Structured summary of trial design, methods, results, conclusions | pp. 3–4, l. 62–97 (structured abstract) |
| **Introduction** | | |
| **2a** | Scientific background; rationale for cluster design and SW design | pp. 5–7, section 1; p. 8, l. 200–201 (rationale for stepped-wedge design); p. 22, section 4.2 |
| **2b** | Specific objectives or hypotheses | p. 7, l. 178–189 |
| **Methods** | | |
| **3a** | Description and diagram: cluster definition, sequences, clusters/sequence, periods, step duration, participants | p. 8, section 2, l. 196–208; p. 13, Figure 1 (l. 356) |
| **3b** | Important changes to methods after commencement, with reasons | p. 9, l. 231–233 (Level II sites not recruited); p. 11, l. 303–308 |
| **4a** | Eligibility criteria for clusters and participants | p. 9, section 2.1, l. 229–241 |
| **4b** | Settings and locations where data were collected | p. 9, section 2.1, l. 229–230; Acknowledgements (consortium, p. 26); Additional file 4 |
| **5** | Intervention and control conditions with sufficient detail for replication | pp. 9–10, section 2.3, l. 253–270; p. 10, l. 258–259 (control condition); Additional file 2 |
| **6a** | Pre-specified primary and secondary outcome measures | pp. 10–11, section 2.4, l. 272–308; Table 1 (p. 11, l. 310) |
| **6b** | Changes to trial outcomes after commencement, with reasons | p. 11, l. 303–308 (registration, operationalisation of registered outcome constructs, no post-commencement changes) |
| **7a** | Sample size determination with ICC assumption | p. 11, section 2.5, l. 312–318 |
| **7b** | Interim analyses and stopping guidelines | p. 11, l. 317–318 (no interim analyses, no stopping guidelines) |
| **Randomisation** | | |
| **8a** | Method used to generate random allocation to sequences | p. 9, section 2.2, l. 243–244 (independent statistician, computer-generated) |
| **8b** | Type of randomisation; constrained/stratification | p. 9, section 2.2, l. 243–244 (three sequences, 1:1:1, five NICUs per sequence) |
| **9** | Allocation concealment mechanism based on clusters | p. 9, section 2.2, l. 245–246 |
| **10a** | Who generated randomisation, enrolled clusters, assigned to sequences | p. 9, section 2.2, l. 243–244 |
| **10b** | Mechanism for including individual participants in clusters | p. 9, section 2.1, l. 234–235 (consecutive enrolment) |
| **10c** | Consent: from whom, when, for what | p. 9, section 2.1, l. 238–241; pp. 23–24, Declarations, l. 648–660 |
| **11a** | Blinding: who was blinded, how | p. 9, section 2.2, l. 247–252 |
| **11b** | Similarity of treatments | p. 10, section 2.3, l. 258–259 and l. 264–268 |
| **12a** | Statistical methods: time effects, clustering, repeated measures | pp. 11–12, section 2.6, l. 320–342 |
| **12b** | Additional analyses: subgroup, sensitivity, adjusted | p. 12, section 2.6, l. 326–335 |
| **Results** | | |
| **13a** | Participant flow: numbers assessed, assigned, received treatment, analysed | pp. 13–14, section 3.1, l. 351–368; Figure 1 (l. 356) |
| **13b** | Losses and exclusions with reasons | p. 13, section 3.1; p. 12, l. 338–339 (32 cases excluded, reason stated) |
| **14a** | Dates defining steps, intervention initiation, deviations | p. 8, l. 216–217 (April 2022 – March 2024, four periods at 6-month intervals, two-month follow-up) |
| **14b** | Why the trial ended or was stopped | Completed as planned |
| **15** | Baseline characteristics by treatment condition or sequence | p. 14, Table 2 (l. 370); Additional file 4 (by period and sequence) |
| **16** | Number of observations/clusters per analysis | pp. 14–15, Table 3 (l. 394); Additional file 5 |
| **17a** | Results for each outcome: effect size, precision, correlations, time effects | pp. 14–16, sections 3.2–3.3, l. 377–443; Figures 2–3; Additional file 5 |
| **17b** | Binary outcomes: both absolute and relative effect sizes | pp. 14–16, sections 3.2–3.3 (primarily absolute effects); Additional file 5 |
| **18** | Ancillary analyses: subgroup, adjusted, pre-specified vs. exploratory | pp. 14–16, sections 3.2–3.3 (subgroups, sensitivity analyses); p. 15, l. 400–401 (no adjustment for multiple testing) |
| **19** | Important harms or unintended effects | p. 10, l. 288–290 (ascertainment); p. 15, l. 410–412 (results); pp. 19–20, l. 518–521 |
| **Discussion** | | |
| **20** | Limitations: bias, imprecision, multiplicity | p. 22, section 4.2, l. 590–610 |
| **21** | Generalisability: clusters and/or individual participants | p. 22, section 4.2, l. 590–610; Additional file 4 |
| **22** | Interpretation consistent with results, balancing benefits and harms | pp. 19–23, sections 4.1–4.3 and section 5, l. 485–639 |
| **Other Information** | | |
| **23** | Registration number and registry | p. 4, l. 99 (abstract); p. 8, l. 218–220 (DRKS00025058, with URL and date of registration) |
| **24** | Where full protocol can be accessed | p. 8, l. 224–225 (protocol, ref [32]); p. 9, l. 226–227 (statistical analysis plan) |
| **25** | Sources of funding, role of funders | p. 25, l. 703–707 (Innovation Fund, 01NVF19027) |
| **26** | Ethics review, consent, justification for waivers | p. 8, l. 218–219 (ethics approval 21-1506); pp. 23–24, Declarations, l. 648–660 |

## Table 3: CONSORT 2025 Checklist

*Hopewell et al., BMJ 2025;389:e081123. The CONSORT extension for stepped-wedge cluster randomised trials (Table 2) has not yet been updated to the CONSORT 2025 checklist; both instruments are therefore provided.*

| **Item** | **Checklist Item** | **Location (Page / Line)** |
| --- | --- | --- |
| **Title and abstract** | | |
| **1a** | Identification as a randomised trial | p. 1 (title) |
| **1b** | Structured summary of the trial design, methods, results, and conclusions | pp. 3–4, l. 62–97 |
| **Open science** | | |
| **2** | Name of trial registry, identifying number (with URL) and date of registration | p. 8, l. 218–220; p. 11, l. 303–304; p. 4, l. 99 (abstract) |
| **3** | Where the trial protocol and statistical analysis plan can be accessed | p. 8, l. 224–225 (protocol, ref [32]); p. 9, l. 226–227 (statistical analysis plan) |
| **4** | Where and how the individual de-identified participant data (including data dictionary), statistical code and any other materials can be accessed | pp. 24–25, Data Availability, l. 662–696; Additional file 6 (codebook); dataset ref [78] |
| **5a** | Sources of funding and other support, and role of funders in the design, conduct, analysis and reporting of the trial | p. 25, l. 703–707 |
| **5b** | Financial and other conflicts of interest of the manuscript authors | p. 25, l. 699–700 |
| **Introduction** | | |
| **6** | Scientific background and rationale | pp. 5–7, section 1, l. 106–177 |
| **7** | Specific objectives related to benefits and harms | p. 7, l. 178–189 |
| **Methods** | | |
| **8** | Details of patient or public involvement in the design, conduct and reporting of the trial | p. 10, section 2.3, l. 268–270 |
| **9** | Description of trial design including type of trial, allocation ratio, and framework | p. 8, l. 196–208; p. 9, l. 243–244 (1:1:1) |
| **10** | Important changes to the trial after it commenced including any outcomes or analyses that were not prespecified, with reason | p. 11, l. 303–308; p. 9, l. 231–233 (Level II sites not recruited) |
| **11** | Settings and locations where the trial was conducted | p. 9, l. 229–233 |
| **12a** | Eligibility criteria for participants | p. 9, l. 234–241 |
| **12b** | If applicable, eligibility criteria for sites and for individuals delivering the interventions | p. 9, l. 229–230 |
| **13** | Intervention and comparator with sufficient details to allow replication; where additional materials describing the intervention and comparator can be accessed | pp. 9–10, section 2.3, l. 253–270; Additional file 2 (TIDieR, Proctor) |
| **14** | Prespecified primary and secondary outcomes, including the specific measurement variable, analysis metric, method of aggregation, and time point for each outcome | pp. 10–11, section 2.4, l. 272–308; Table 1 (p. 11, l. 310) |
| **15** | How harms were defined and assessed (eg, systematically, non-systematically) | p. 10, l. 288–290 |
| **16a** | How sample size was determined, including all assumptions supporting the sample size calculation | p. 11, section 2.5, l. 312–318 |
| **16b** | Explanation of any interim analyses and stopping guidelines | p. 11, l. 317–318 |
| **17a** | Who generated the random allocation sequence and the method used | p. 9, l. 243–244 |
| **17b** | Type of randomisation and details of any restriction | p. 9, l. 243–244 |
| **18** | Mechanism used to implement the random allocation sequence, describing any steps to conceal the sequence until interventions were assigned | p. 9, l. 245–246 |
| **19** | Whether the personnel who enrolled and those who assigned participants to the interventions had access to the random allocation sequence | p. 9, l. 243–246 |
| **20a** | Who was blinded after assignment to interventions | p. 9, l. 247–252 |
| **20b** | If blinded, how blinding was achieved and description of the similarity of interventions | p. 9, l. 247–252 |
| **21a** | Statistical methods used to compare groups for primary and secondary outcomes, including harms | pp. 11–12, section 2.6, l. 320–325 |
| **21b** | Definition of who is included in each analysis, and in which group | p. 11, l. 320; p. 12, l. 335–341 |
| **21c** | How missing data were handled in the analysis | p. 12, l. 336–341 |
| **21d** | Methods for any additional analyses, distinguishing prespecified from post hoc | p. 12, section 2.6, l. 326–335 |
| **Results** | | |
| **22a** | For each group, the numbers of participants who were randomly assigned, received intended intervention, and were analysed for the primary outcome | pp. 13–14, section 3.1, l. 351–368; Figure 1 (l. 356) |
| **22b** | For each group, losses and exclusions after randomisation, together with reasons | p. 13, section 3.1; p. 12, l. 338–339 (32 cases excluded, reason stated) |
| **23a** | Dates defining the periods of recruitment and follow-up for outcomes of benefits and harms | p. 8, l. 216–217 |
| **23b** | If relevant, why the trial ended or was stopped | Not applicable — the trial was completed as planned |
| **24a** | Intervention and comparator as they were actually administered, including who delivered them, adherence and fidelity | pp. 16–17, section 3.4, l. 444–472; Additional file 4 |
| **24b** | Concomitant care received during the trial for each group | p. 10, l. 258–259 (control condition: standard NICU care) |
| **25** | A table showing baseline demographic and clinical characteristics for each group | p. 14, Table 2 (l. 370); Additional file 4 (Tables 1–2) |
| **26** | For each primary and secondary outcome, by group: number analysed, number with available data, result and estimated effect size with its precision, and for binary outcomes both absolute and relative effect size | pp. 14–16, sections 3.2–3.3, l. 377–443; Table 3 (l. 394); Figures 2–3; Additional file 5 |
| **27** | All harms or unintended events in each group | p. 15, l. 410–412; pp. 19–20, l. 518–521 |
| **28** | Any other analyses performed, including subgroup and sensitivity analyses, distinguishing prespecified from post hoc | pp. 14–16, sections 3.2–3.3; p. 12, l. 326–335; Additional file 5 |
| **Discussion** | | |
| **29** | Interpretation consistent with results, balancing benefits and harms, and considering other relevant evidence | pp. 19–21, section 4.1, l. 485–588 |
| **30** | Trial limitations, addressing sources of potential bias, imprecision, generalisability, and, if relevant, multiplicity of analyses | p. 22, section 4.2, l. 590–610; p. 15, l. 400–401 (no adjustment for multiple testing) |
